# A benchmark study of ab initio gene prediction methods in diverse eukaryotic organisms

**DOI:** 10.1186/s12864-020-6707-9

**Published:** 2020-04-09

**Authors:** Nicolas Scalzitti, Anne Jeannin-Girardon, Pierre Collet, Olivier Poch, Julie D. Thompson

**Affiliations:** Department of Computer Science, ICube, CNRS, University of Strasbourg, Strasbourg, France

**Keywords:** Genome annotation, Gene prediction, Protein prediction, Benchmark study

## Abstract

**Background:**

The draft genome assemblies produced by new sequencing technologies present important challenges for automatic gene prediction pipelines, leading to less accurate gene models. New benchmark methods are needed to evaluate the accuracy of gene prediction methods in the face of incomplete genome assemblies, low genome coverage and quality, complex gene structures, or a lack of suitable sequences for evidence-based annotations.

**Results:**

We describe the construction of a new benchmark, called G3PO (benchmark for Gene and Protein Prediction PrOgrams), designed to represent many of the typical challenges faced by current genome annotation projects. The benchmark is based on a carefully validated and curated set of real eukaryotic genes from 147 phylogenetically disperse organisms, and a number of test sets are defined to evaluate the effects of different features, including genome sequence quality, gene structure complexity, protein length, etc. We used the benchmark to perform an independent comparative analysis of the most widely used ab initio gene prediction programs and identified the main strengths and weaknesses of the programs. More importantly, we highlight a number of features that could be exploited in order to improve the accuracy of current prediction tools.

**Conclusions:**

The experiments showed that ab initio gene structure prediction is a very challenging task, which should be further investigated. We believe that the baseline results associated with the complex gene test sets in G3PO provide useful guidelines for future studies.

## Background

The plunging costs of DNA sequencing [1] have made de novo genome sequencing widely accessible for an increasingly broad range of study systems with important applications in agriculture, ecology, and biotechnologies amongst others [2]. The major bottleneck is now the high-throughput analysis and exploitation of the resulting sequence data [3]. The first essential step in the analysis process is to identify the functional elements, and in particular the protein-coding genes. However, identifying genes in a newly assembled genome is challenging, especially in eukaryotes where the aim is to establish accurate gene models with precise exon-intron structures of all genes [3–5].

Experimental data from high-throughput expression profiling experiments, such as RNA-seq or direct RNA sequencing technologies, have been applied to complement the genome sequencing and provide direct evidence of expressed genes [6, 7]. In addition, information from closely related genomes can be exploited, in order to transfer known gene models to the target genome. Numerous automated gene prediction methods have been developed that incorporate similarity information, either from transcriptome data or known gene models, including GenomeScan [8], GeneWise [9], FGENESH [10], Augustus [11], Splign [12], CodingQuarry [13], and LoReAN [14].

The main limitation of similarity-based approaches is in cases where transcriptome sequences or closely related genomes are not available. Furthermore, such approaches encourage the propagation of erroneous annotations across genomes and cannot be used to discover novelty [5]. Therefore, similarity-based approaches are generally combined with ab initio methods that predict protein coding potential based on the target genome alone. Ab initio methods typically use statistical models, such as Support Vector Machines (SVMs) or hidden Markov models (HMMs), to combine two types of sensors: signal and content sensors. Signal sensors exploit specific sites and patterns such as splicing sites, promotor and terminator sequences, polyadenylation signals or branch points. Content sensors exploit the coding versus non-coding sequence features, such as exon or intron lengths or nucleotide composition [15]. Ab initio gene predictors, such as Genscan [16], GlimmerHMM [17], GeneID [18], FGENESH [10], Snap [19], Augustus [20], and GeneMark-ES [21], can thus be used to identify previously unknown genes or genes that have evolved beyond the limits of similarity-based approaches.

Unfortunately, automatic ab initio gene prediction algorithms often make substantial errors and can jeopardize subsequent analyses, including functional annotations, identification of genes involved in important biological process, evolutionary studies, etc. [22–25]. This is especially true in the case of large “draft” genomes, where the researcher is generally faced with an incomplete genome assembly, low coverage, low quality, and high complexity of the gene structures. Typical errors in the resulting gene models include missing exons, non-coding sequence retention in exons, fragmenting genes and merging neighboring genes. Furthermore, the annotation errors are often propagated between species and the more “draft” genomes we produce, the more errors we create and propagate [3–5]. Other important challenges that have attracted interest recently include the prediction of small proteins/peptides coded by short open reading frames (sORFs) [26, 27] or the identification of events such as stop codon recoding [28]. These atypical proteins are often overlooked by the standard gene prediction pipelines, and their annotation requires dedicated methods or manual curation.

The increased complexity of today’s genome annotation process means that it is timely to perform an extensive benchmark study of the main computational methods employed, in order to obtain a more detailed knowledge of their advantages and disadvantages in different situations. Some previous studies have been performed to evaluate the performance of the most widely used ab initio gene predictors. One of the first studies [29] compared 9 programs on a set of 570 vertebrate sequences encoding a single functional protein, and concluded that most of the methods were overly dependent on the original set of sequences used to train the gene models. More recent studies have focused on gene prediction in specific genomes, usually from model or closely-related organisms, such as mammals [30], human [31, 32] or eukaryotic pathogen genomes [33], since they have been widely studied and many gene structures are available that have been validated experimentally. To the best of our knowledge, no recent benchmark study has been performed on complex gene sequences from a wide range of organisms.

Here, we describe the construction of a new benchmark, called G3PO – benchmark for Gene and Protein Prediction PrOgrams, containing a large set of complex eukaryote genes from very diverse organisms (from human to protists). The benchmark consists of 1793 reference genes and their corresponding protein sequences from 147 species and covers a range of gene structures from single exon genes to genes with over 20 exons. A crucial factor in the design of any benchmark is the quality of the data included. Therefore, in order to ensure the quality of the benchmark proteins, we constructed high quality multiple sequence alignments (MSA) and identified the proteins with inconsistent sequence segments that might indicate potential sequence annotation errors. Protein sequences with no identified errors were labeled ‘Confirmed’, while sequences with at least one error were labeled ‘Unconfirmed’. The benchmark thus contains both Confirmed and Unconfirmed proteins (defined in Methods: Benchmark test sets) and represents many of the typical prediction errors presented above. We believe the benchmark allows a realistic evaluation of the currently available gene prediction tools on challenging data sets.

We used the G3PO benchmark to compare the accuracy and efficiency of five widely used ab initio gene prediction programs, namely Genscan, GlimmerHMM, GeneID, Snap and Augustus. Our initial comparison highlighted the difficult nature of the test cases in the G3PO benchmark, since 68% of the exons and 69% of the Confirmed protein sequences were not predicted with 100% accuracy by all five gene prediction programs. Different benchmark tests were then designed in order to identify the main strengths and weaknesses of the different programs, but also to investigate the impact of the genomic environment, the complexity of the gene structure, or the nature of the final protein product on the prediction accuracy.

## Results

The presentation of the results is divided into 3 sections, describing (i) the data sets included in the G3PO benchmark, (ii) the overall prediction quality of the five gene prediction programs tested and (iii) the effects of various factors on gene prediction quality.

### Benchmark data sets

The G3PO benchmark contains 1793 proteins from a diverse set of organisms (Additional file 1: Table S1), which can be used for the evaluation of gene prediction programs. The proteins were extracted from the Uniprot [34] database, and are divided into 20 orthologous families (called BBS1–21, excluding BBS14) that are representative of complex proteins, with multiple functional domains, repeats and low complexity regions (Additional file 1: Table S2). The benchmark test sets cover many typical gene prediction tasks, with different gene lengths, protein lengths and levels of complexity in terms of number of exons (Additional file 1: Fig. S1). For each of the 1793 proteins, we identified the corresponding genomic sequence and the exon map in the Ensembl [35] database. We also extracted the same genomic sequences with additional DNA regions ranging from 150 to 10,000 nucleotides upstream and downstream of the gene, in order to represent more realistic genome annotation tasks. Additional file 1: Fig. S2 shows the distribution of various features of the 1793 benchmark test cases, at the genome level (gene length, GC content), gene structure level (number and length of exons, intron length), and protein level (length of main protein product).

#### Phylogenetic distribution of benchmark sequences

**Fig. 1:**
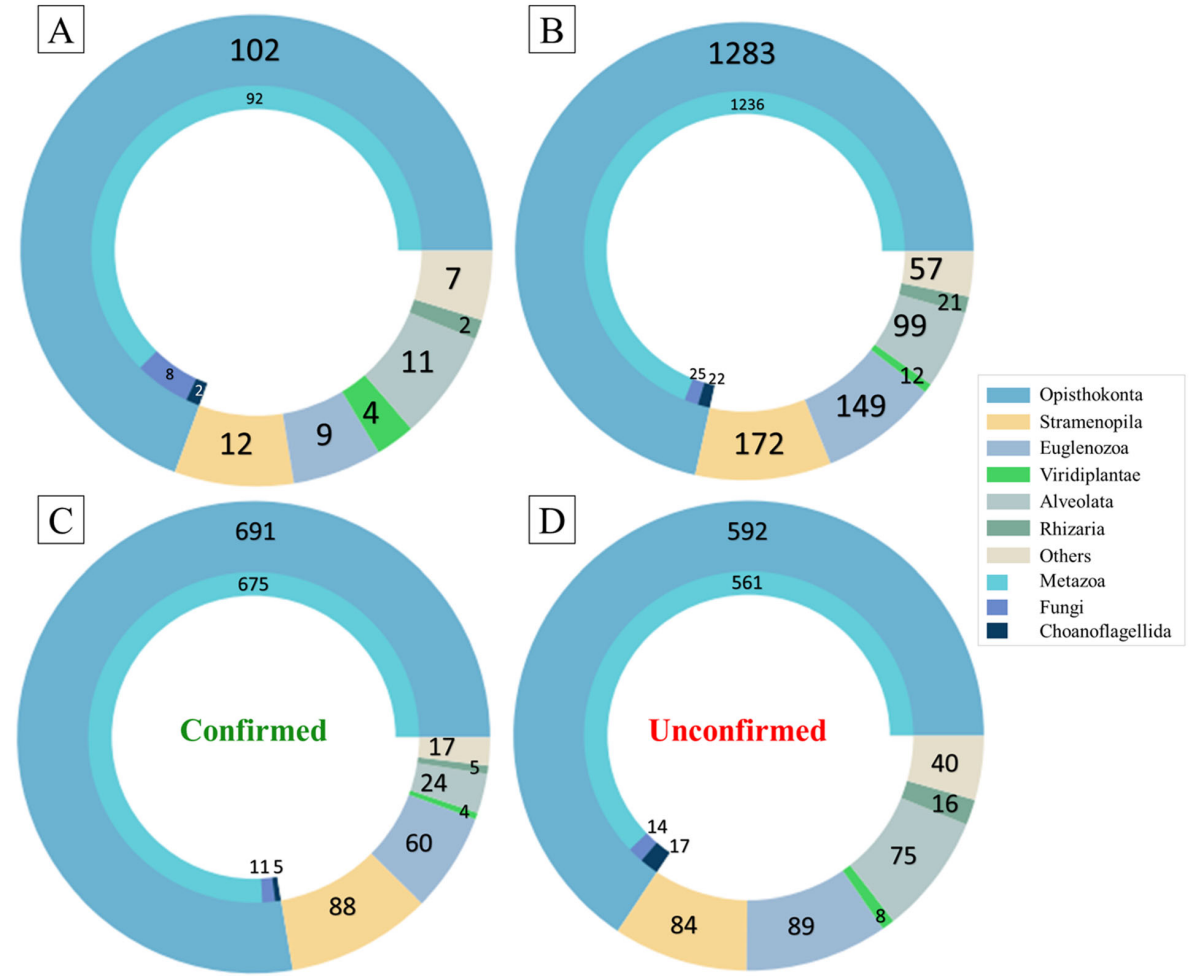
Phylogenetic distribution of the 1793 test cases in the G3PO benchmark. **a** Number of species in each clade. **b** Number of sequences in each clade. **c** Number of sequences in each clade in the Confirmed test set. **d** Number of sequences in each clade in the Unconfirmed test set. The ‘Others’ group corresponds to: Apusozoa, Cryptophyta, Diplomonadida, Haptophyceae, Heterolobosea, Parabasalia

The protein sequences used in the construction of the G3PO benchmark were identified in 147 phylogenetically diverse eukaryotic organisms, ranging from human to protists (Fig. 1a and Additional file 1: Table S3). The majority (72%) of the proteins are from the Opisthokonta clade, which includes 1236 (96.4%) Metazoa, 25 (1.9%) Fungi and 22 (1.7%) Choanoflagellida sequences (Fig. 1b). The next largest groups represented in the database are the Stramenopila (172), Euglenozoa (149) and Alveolata (99) sequences. More divergent species are included in the ‘Others’ group, containing 57 sequences from 6 different clades, namely Apusozoa, Cryptophyta, Diplomonadida, Haptophyceae, Heterolobosea and Parabasalia.

#### Exon map complexity

**Fig. 2:**
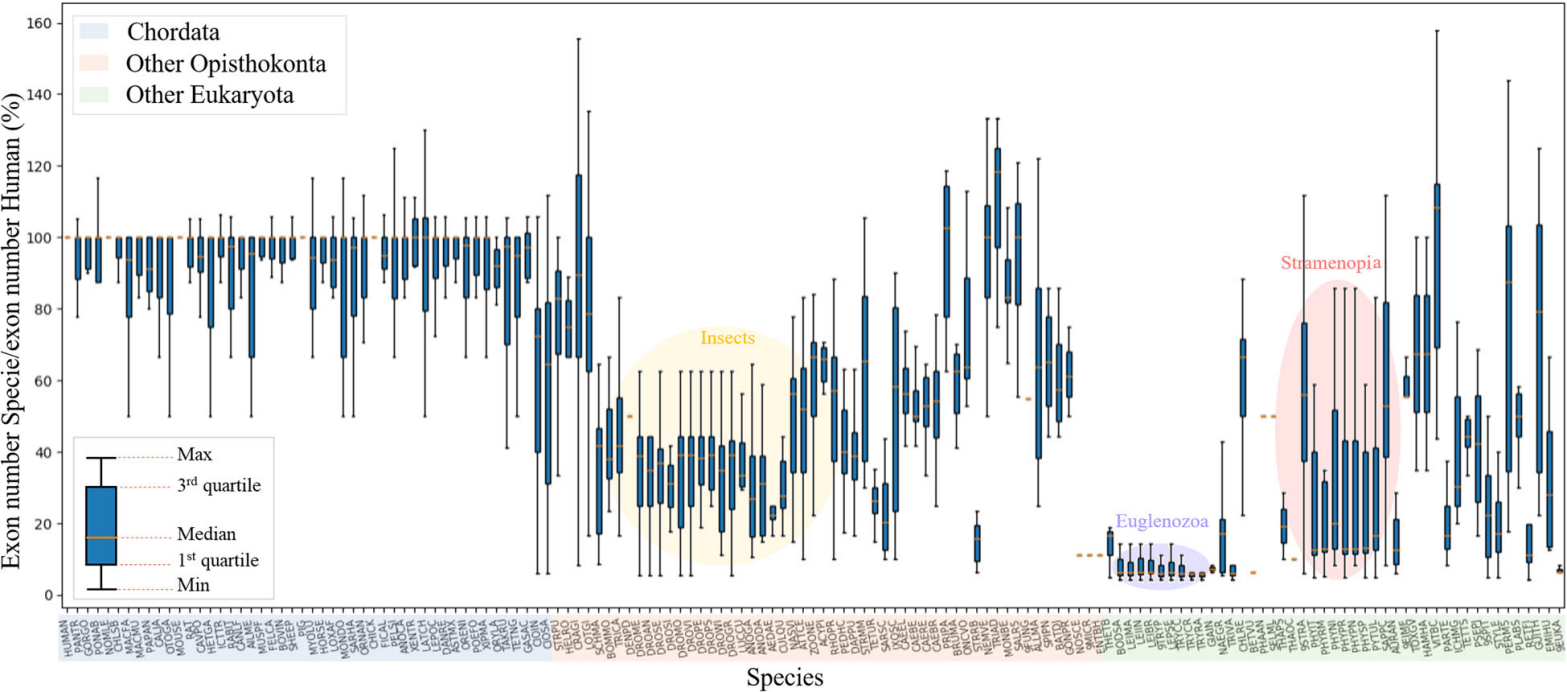
Exon map complexity for each species. Each box plot represents the distribution of the ratio of the number of exons in the gene of a given species (Exon Number Species), to the number of exons in the orthologous human gene (Exon number Human), for all genes in the benchmark. Notable clades include Insects (BOMMO to PEDHC), Euglenozoa (BODSA to TRYRA) or Stramenopila (THAPS to AURAN)

The benchmark was designed to cover a wide range of test cases with different exon map complexities, as encountered in a realistic complete genome annotation project. The test cases in the benchmark range from single exon genes to genes with 40 exons (Additional file 1: Fig. S2). In particular, the different species included in the benchmark present different challenges for gene prediction programs. To illustrate this point, we compared the number of exons in the human genes to the number of exons in the orthologous genes from each species (Fig. 2). Three main groups can be distinguished: i) Chordata, ii) other Opisthokonta (Mollusca, Platyhelminthes, Panarthropoda, Nematoda, Cnidaria, Fungi and Choanoflagellida) and iii) other Eukaryota (Amoebozoa, Euglenozoa, Heterolobosza, Parabasalia, Rhodophyta, Viridiplantae, Stramenopila, Alveolata, Rhizaria, Cryptophyta, Haptophyceae). As might be expected, the sequences in the Chordata group generally have a similar number of exons compared to the Human sequences. The sequences in the ‘other Opisthokonta’ group have greater heterogeneity, as expected due to their phylogenetic divergence, although some classes, such as the insects are more homogeneous. The genes in this group have three times fewer exons on average, compared to the Chordata group. The ‘other Eukaryota’ group includes diverse clades ranging from Viridiplantae and Protists, although the exon map complexity is relatively homogeneous within each clade. For example, in the Euglenozoa clades, all sequences have less than 20% of the number of exons compared to human.

#### Quality of protein sequences

**Fig. 3:**
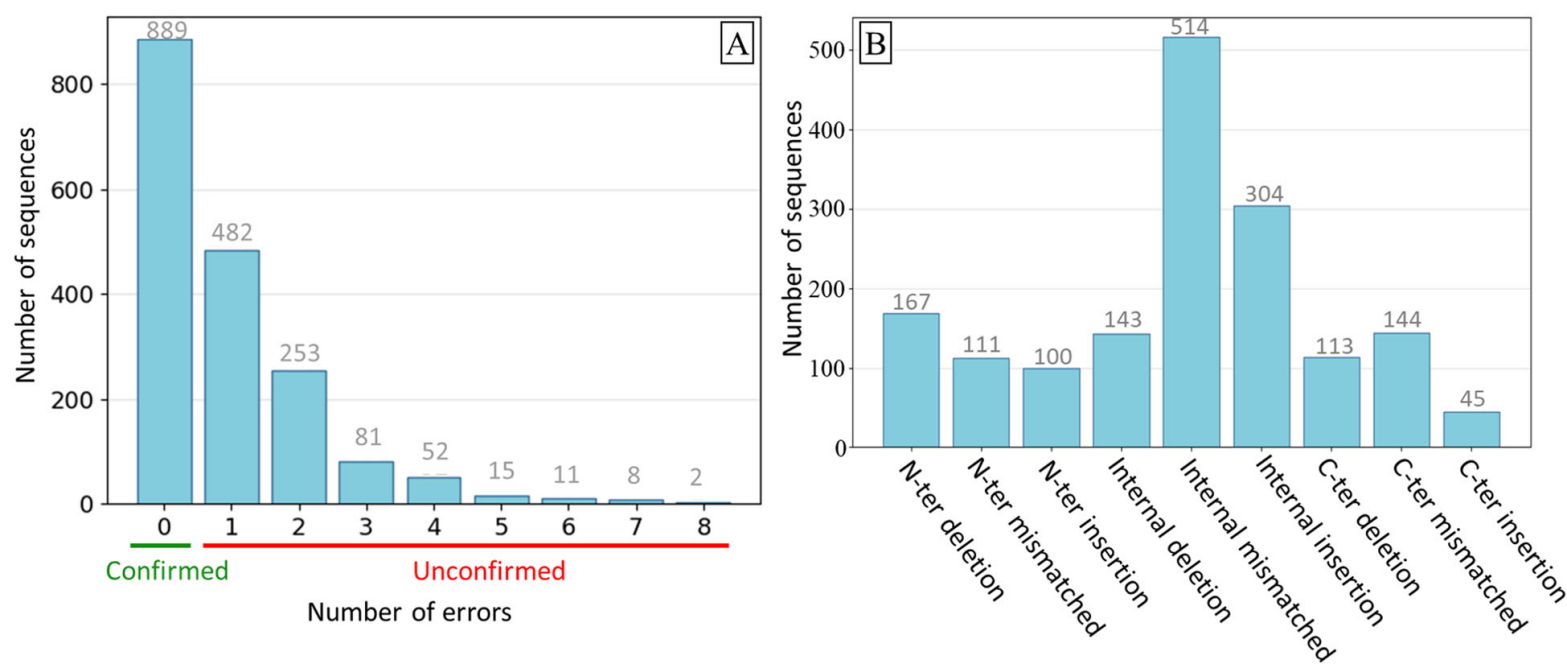
**a** Number of identified sequence errors in the 1793 benchmark proteins. **b** Number of ‘Unconfirmed’ protein sequences for each error category

The protein sequences included in the benchmark were extracted from the public databases, and it has been shown previously that these resources contain many sequence errors [22–25]. Therefore, we evaluated the quality of the protein sequences in G3PO using a homology-based approach (see Methods), similar to that used in the GeneValidator program [23]. We thus identified protein sequences containing potential errors, such as inconsistent insertions/deletions or mismatched sequence segments (Additional file 1: Fig. S3 and Methods). Of the 1793 proteins, 889 (49.58%) protein sequences had no identified errors and were classified as ‘Confirmed’, while 904 (50.42%) protein sequences had from 1 to 8 potential errors (Fig. 3a) and were classified as ‘Unconfirmed’. The 904 Unconfirmed sequences contain a total of 1641 errors, i.e. each sequence has an average of 1.8 errors. Additional file 1: Table S4 shows the number of Unconfirmed sequences and the total number of errors identified for each species included in the benchmark. We further characterized the Unconfirmed sequences by the categories of error they contain (Fig. 3b) and by orthologous protein family (Additional file 1: Fig. S4A and B). All the protein families contain Unconfirmed sequences, regardless of the number or length of the sequences, although the ratio of Confirmed to Unconfirmed sequences is not the same in all families. For example, the BBS6, 11, 12, 18 families, that are present mainly in vertebrate species, have more Confirmed sequences (68.5, 80.0, 52.3, 61.1% respectively). Inversely, the majority of sequences in the BBS8 and 9 families, that contain many phylogenetically disperse organisms, are Unconfirmed (68.8, 73.3% respectively). The majority of the 1641 errors (58.4%) are internal (i.e. do not affect the N- or C-termini) and 31% are internal mismatched segments, while N-terminal errors (378 = 23.0%) are more frequent than C-terminal errors (302 = 18.4%). At the N- and C-termini, deletions are more frequent than insertions (280 and 145, respectively), in contrast to the internal errors, where insertions are more frequent (304 compared to 143).

The distributions of various features are compared for the sets of 889 Confirmed and 904 Unconfirmed sequences in Additional file 1: Fig. S2. There are no significant differences in gene length (*p*-value = 0.735), GC content (p-value = 0.790), number of exons (p-value = 0.073), and exon/intron lengths (p-value = 0.690 / p-value = 0.949) between the Confirmed and Unconfirmed sequences. The biggest difference is observed at the protein level, where the Confirmed protein sequences are 13% shorter than the Unconfirmed proteins (p-value = 8.75 × 10^− 9^). We also compared the phylogenetic distributions observed in the Confirmed and Unconfirmed sequence sets (Fig. 1c and d). Two clades had a higher proportion of Confirmed sequences, namely Opisthokonta (691/1283 = 54%) and Stramenopila (88/172 = 51%). In contrast, Alveolata (24/99 = 24%), Rhizaria (5/21 = 24%) and Choanoflagellida (5/22 = 22%) had fewer Confirmed than Unconfirmed sequences.

#### Quality of genome sequences

The genomic sequences corresponding to the reference proteins in G3PO were extracted from the Ensembl database. In all cases, the soft mask option was used (see Methods) to localize repeated or low complexity regions. However, some sequences still contained undetermined nucleotides, represented by ‘n’ characters, probably due to genome sequencing errors or gaps in the assembly. Undetermined (UDT) nucleotides were found in 283 (15.8%) genomic sequences from 58 (39.5%) organisms, of which 281 sequences (56 organisms) were from the metazoan clade (Additional file 1: Fig. S5). Of these 283 sequences, 133 were classified as Confirmed and 150 were classified as Unconfirmed.

We observed important differences between the characteristics of the sequences with UDT regions and the other G3PO sequences, for both Confirmed and Unconfirmed proteins (Additional file 1: Table S5). The average length of the 283 gene sequences with UDT regions (95,584 nucleotides) is 6 times longer than the average length of the 1510 genes without UDT (15,934 nucleotides), although the protein sequences have similar average lengths (551 amino acids for UDT sequences compared to 514 amino acids for non UDT sequences). Sequences with UDT regions have twice as many exons, three times shorter exons and five times longer introns than sequences without UDT.

#### Evaluation metrics

The benchmark includes a number of different performance metrics that are designed to measure the quality of the gene prediction programs at different levels. At the nucleotide level, we study the ability of the programs to correctly classify individual nucleotides found within exons or introns. At the exon level, we applied a strict definition of correctly predicted exons: the boundaries of the predicted exons should exactly match the boundaries of the benchmark exons. At the protein level, we compare the predicted protein to the benchmark sequence and calculate the percent sequence identity (defined as the number of identical amino acids compared to the number of amino acids in the benchmark sequence). It should be noted that, due to their strict definition, scores at the exon level are generally lower. For example, in some cases, the predicted exon boundary may be shifted by a few nucleotides, resulting in a low exon score but high nucleotide and protein level scores.

### Evaluation of gene prediction programs

**Fig. 4:**
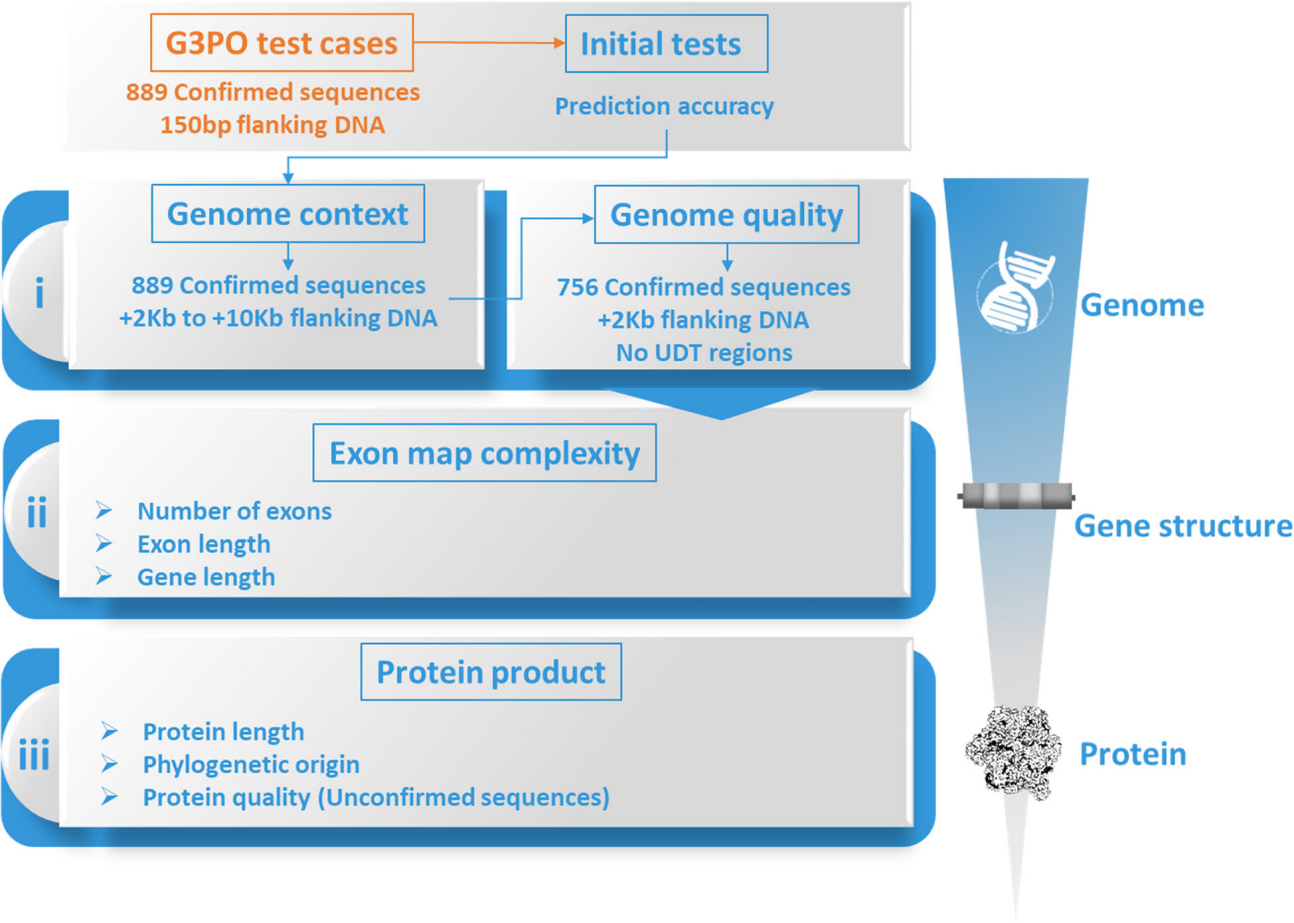
Workflow of different tests performed to evaluate gene prediction accuracy. The initial tests are based on the 889 confirmed proteins and their genomic sequences corresponding to the gene region with 150 bp flanking sequences. At the genome level, effect of genome context and genome quality are tested, and 756 confirmed sequences with +2Kb flanking sequences and no undetermined (UDT) regions are selected. These are used at the gene structure and protein levels, to investigate effects of factors linked to exon map complexity and the final protein product

We selected five widely used gene prediction programs: Augustus, Genscan, GeneID, GlimmerHMM and Snap. These programs all use Hidden Markov Models (HMMs) trained on different sets of known protein sequences and take into account different context sensors, as summarized in Table 1. Each prediction program was run with the default settings, except for the species model to be used. As the benchmark contains sequences from a wide range of species, we selected the most pertinent training model for each sequence, based on their taxonomic proximity (see Methods). The genomic sequences for the 1793 test cases in the G3PO benchmark were used as input to the selected gene prediction programs and a series of tests were performed (outlined in Fig. 4), in order to identify the strong and weak points of the different algorithms, as well as to highlight specific factors affecting prediction accuracy.

**Table 1:** Main characteristics of the gene prediction programs evaluated in this study. GHMM: Generalized hidden Markov model; UTR: Untranslated regions

Gene predictor	Signal sensors	Content sensors	Algorithm model	Organism-specific models
Genscan (version 1.0)	Promoter (15 bp), cap site (8 bp), TATA to cap site distance of 30 to 36 bp, donor (− 3 to + 6 bp)/acceptor (− 20 to + 3) splice sites, polyadenylation, translation start/stop sites	Intergenic, 5′−/3′-UTR, exon/introns in 3 phases, forward/reverse strands	3-periodic fifth-order Markov model (GHMM)	3 models
GlimmerHMM (version 3.02)	Donor (16 bp)/ acceptor (29 bp) splice sites, start/stop codons	Exon/intron in one frame,intron length 50–1500 bp, total coding length > 200 bp	Hidden Markov model (GHMM)	5 models
GeneID (version 1.4)	Donor/acceptor splice sites (− 3 to + 6 bp), start/stop codons	First/initial/last exon, single-exon gene, intron, intron length > 40 bp, intergenic distance > 300 bp	Fifth-order Markov model (HMM)	66 models
SNAP (version 2006-07-28)	Donor (− 3 to + 6 bp) /acceptor (− 24 to + 3) splice sites, translation start (− 6 to + 6 bp) /stop (− 6 to + 3 bp) sites	intergenic, single-exon gene, first/initial/last exon, introns in 3 phases	Fourth-order Markov model (GHMM)	11 models
Augustus (version 3.3.2)	Donor (− 3 to + 6 bp) /acceptor (− 5 to + 1 bp) splice sites, branch point (32 bp), translation start (− 20 to + 3)/stop (3 bp) sites	intergenic, single exon gene, first/initial/last exon, short/long introns in 3 phases and forward/reverse strands, isochore boundaries	Fourth-order Interpolated Markov model (GHMM)	109 models

#### Gene prediction accuracy

**Fig. 5:**
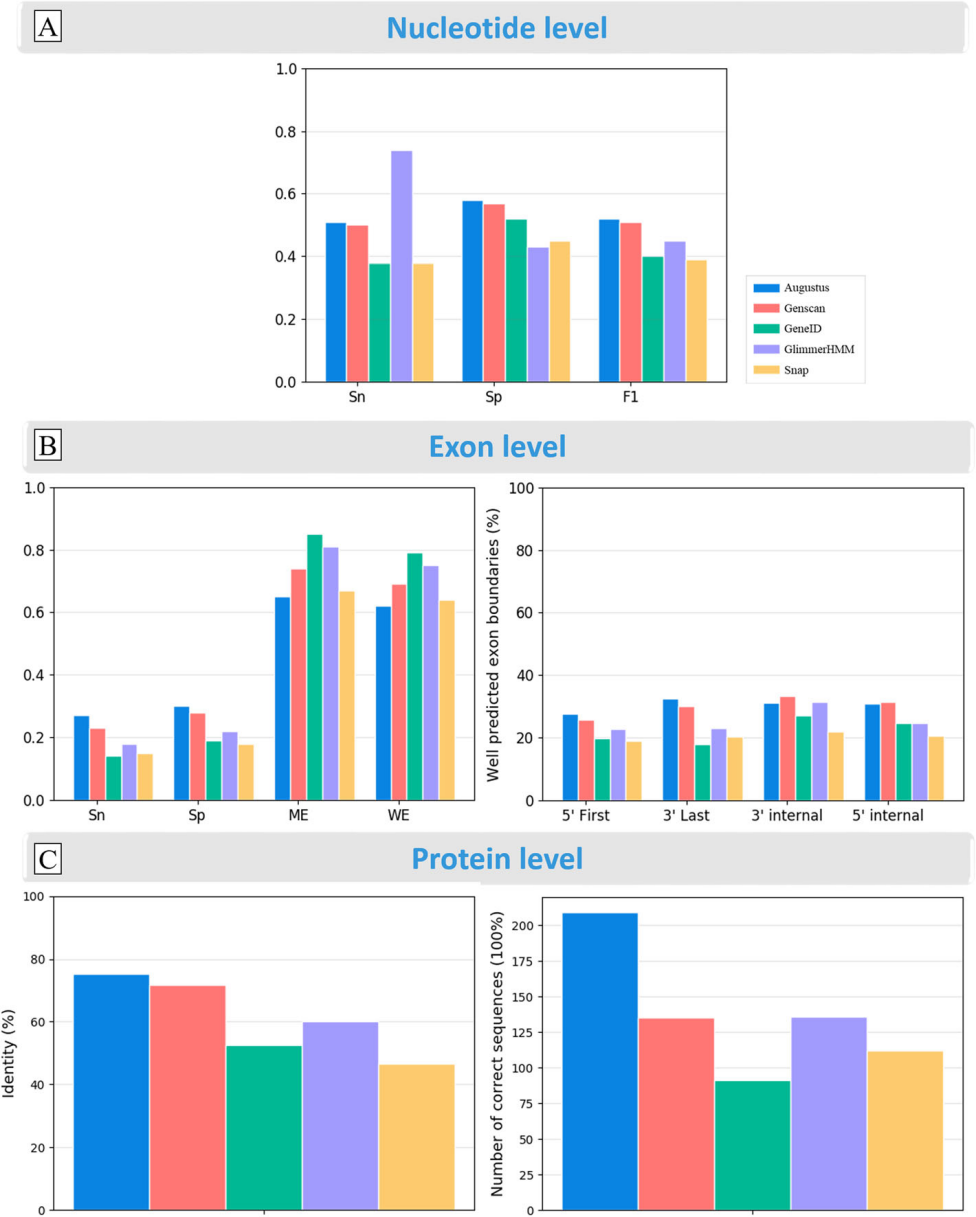
Overall performance of the five gene prediction programs, using the 889 Confirmed sequences with 150 bp flanking sequences, at the **a** nucleotide, **b** exon and **c** protein levels. Sn = sensitivity; Sp = specificity; F1 = F1 score; ME = Missing Exon; WE = Wrong Exon; 5′ First = percentage of correctly predicted 5′ boundaries of first exons only; 3′ Last = percentage of correctly predicted 3′ boundaries of last exons; 3′ and 5′ internal are the percentage of correctly predicted 3′ and 5′ internal exon boundaries. %Identity indicates the average sequence identity observed between the predicted proteins and the Confirmed benchmark sequences

In order to estimate the overall accuracy of the five gene prediction programs, the genes predicted by the programs were compared to the benchmark sequences in G3PO. At this stage, we included only the 889 Confirmed proteins, and used the genomic sequences corresponding to the gene region with 150 bp flanking sequence upstream and downstream of the gene (Fig. 4 – Initial tests) as input. Figure 5(a-c) and Additional file 1: Table S6 show the mean quality scores at different levels: nucleotide, exon structure and final protein sequence (defined in Methods).

At the nucleotide level (Fig. 5a), most of the programs have higher specificities than sensitivities (with the exception of GlimmerHMM), meaning that they tend to underpredict. F1 scores range from 0.39 for Snap to 0.52 for Augustus, meaning that it has the best accuracy.

**Fig. 6:**
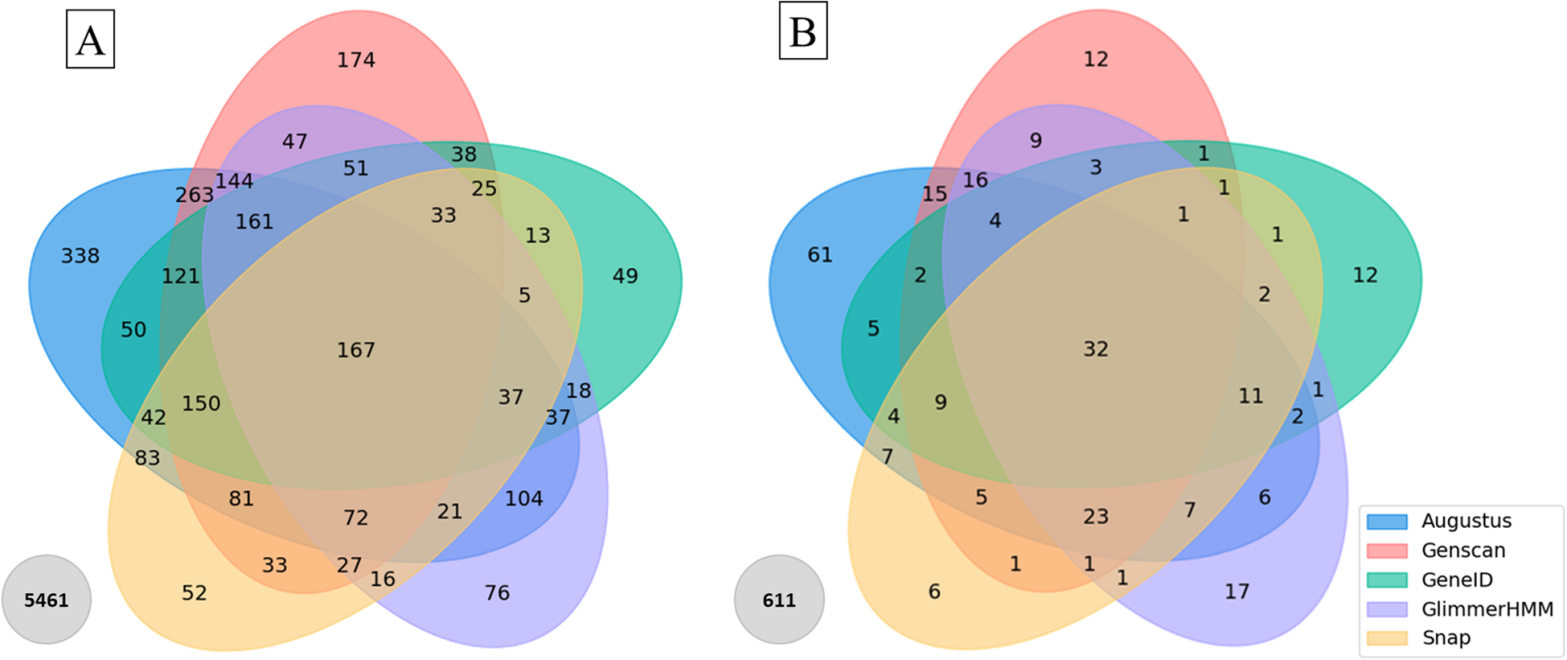
Venn diagrams representing **a** the number of correct exons predicted by each program, and **b** the number of perfectly predicted proteins by each program. The grey circles indicate the number of exons/proteins badly predicted by all programs

At the exon level (Fig. 5b left), Augustus and Genscan achieve higher sensitivities (0.27, 0.23 respectively) and specificities (0.30, 0.28 respectively) than the other programs. Nevertheless, the number of mis-predicted exons remains high with 65 and 74% Missing Exons and 62 and 69% Wrong Exons respectively for Augustus and Genscan. At this level, GeneID and Snap have the lowest sensitivity and specificity, indicating that the predicted splice boundaries are not accurate. We also investigated whether the exon position had an effect on prediction accuracy, by comparing the percentage of well predicted first and last exons with the percentage of well predicted internal exons (Fig. 5b right). The internal exons are predicted better than the first and last exons. In addition, for all exons, the 3′ boundary is generally predicted better than the 5′ boundary. To further investigate the complementarity of the different programs, we plotted the number of Correct Exons (i.e. both 5′ and 3′ exon boundaries correctly predicted) identified by at least one of the programs (Fig. 6a). A total of 167 exons were found by all five programs, suggesting that they are relatively simple to identify. More importantly, 689 exons were correctly predicted by only one program, while 5461 (68.4%) exons were not predicted correctly by any of the programs.

As might be expected, the nucleotide and exon scores are reflected at the protein level (Fig. 5c), with Augustus again achieving the best score, obtaining 75% sequence identity overall and predicting 209 of the 889 (23.5%) Confirmed proteins with 100% accuracy. GeneID and Snap have the lowest scores in terms of perfect protein predictions (52.6, 46.6% respectively). Again, we investigated the complementarity of the programs, by plotting the number of proteins that were perfectly predicted (100% identity) by at least one of the programs (Fig. 6b). Only 32 proteins are perfectly predicted by all five programs, while 108 proteins were predicted with 100% accuracy by a single program. These were mostly predicted by Augustus (61), followed by GlimmerHMM (17). 611 (69%) of the 889 benchmark proteins were not predicted perfectly by any of the programs included in this study.

#### Computational runtime

We also compared the CPU time required for each program to process the benchmark sequences (Additional file 1: Table S7). Using the gene sequences with 150 bp flanking regions (representing a total length of 51,699,512 nucleotides), Augustus required the largest CPU time (1826 s), taking > 3.4 times as long as the second slowest program, namely GlimmerHMM (540 s). GeneID was the fastest program and completed the gene prediction for the 1793 genomic regions, including 10Kb upstream/downstream flanking nucleotides (total length of 86,970,612 nucleotides), in 260 s.

### Analysis of factors affecting gene prediction quality

Based on the results of our initial comparison of gene prediction accuracy, and particularly the complementarity of the programs highlighted in Fig. 6, we decided to investigate further the different factors that may influence the performance of the prediction programs. Figure 4 provides an overview of the different tests performed, including: i) factors associated with the input genomic sequence, ii) factors associated with the gene structure, and iii) factors associated with the protein product.

#### Factors associated with the input genomic sequence

**Fig. 7:**
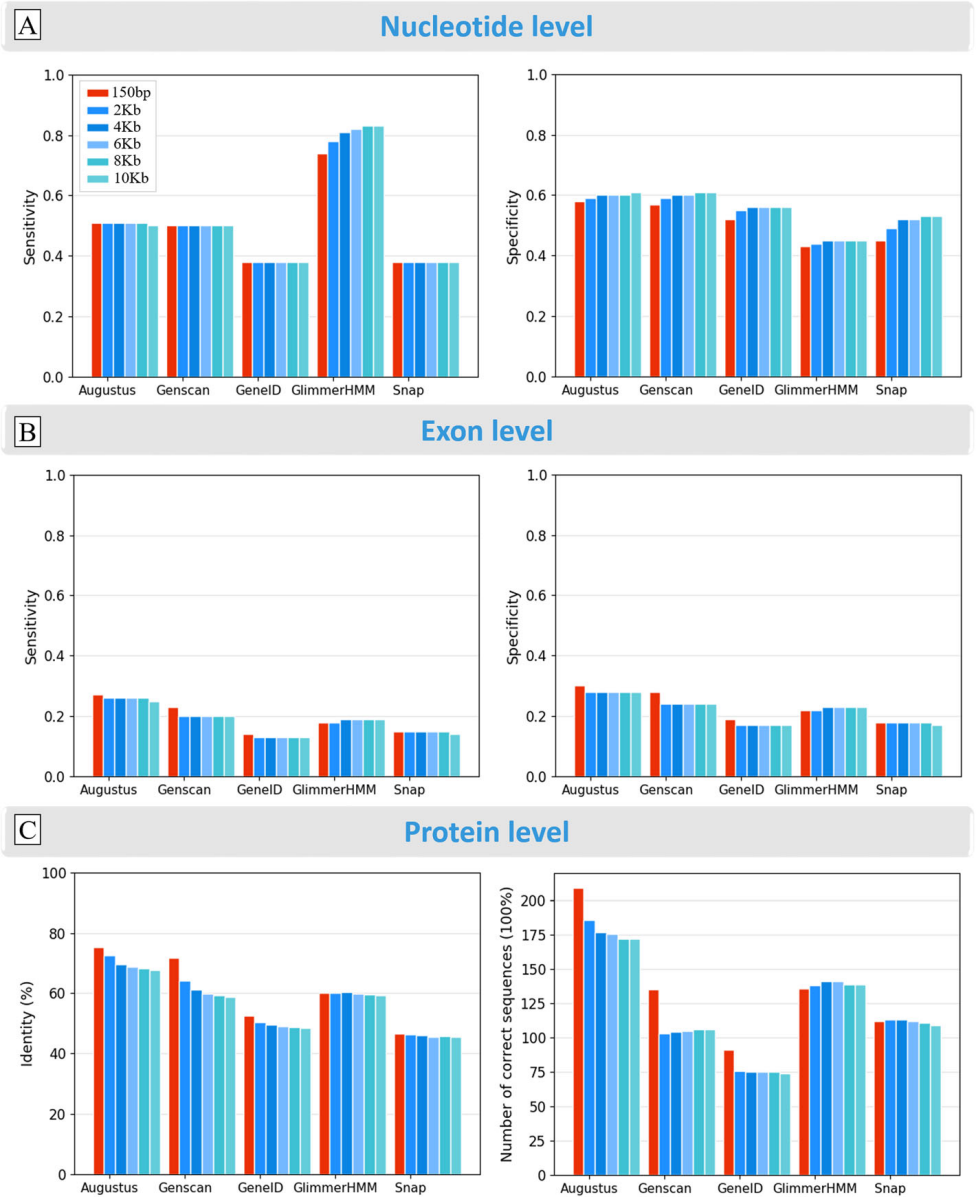
Effect of the genomic context based on the different lengths of upstream/downstream flanking genomic sequences on the performance of the five gene prediction programs. **a** sensitivity and specificity of prediction of coding nucleotides. **b** sensitivity and specificity of exon prediction. **c** accuracy of protein sequence prediction (% identity) and number of proteins correctly predicted with 100% identity

We first evaluated the genome context and the effect of adding flanking sequences upstream and downstream of the benchmark gene sequence used as input to the prediction programs, using the 889 Confirmed benchmark tests. We added different flanking sequence lengths ranging from 150 bp to 10Kb, and calculated the same quality scores as above, at the nucleotide, exon and protein levels (Fig. 7 and Additional file 1: Table S8).

At the nucleotide level, the sensitivity of Augustus, Genscan, GeneID and Snap is not significantly affected by the addition of the flanking sequences. For GlimmerHMM (*p*-value = 4.8 × 10^− 20^), a significant increase in sensitivity is observed when 2Kb flanking sequences are added, compared to the gene sequences with 150 bp only. In terms of specificity, the addition of 2Kb flanking sequences increases significantly the quality of all the programs (Augustus: p-value = 2.87 × 10^− 7^, Genscan: p-value = 1.27 × 10^− 9^, GeneID: p-value = 8.46 × 10^− 5^, GlimmerHMM: p-value = 2.78 × 10^− 7^, Snap: p-value = 1.03 × 10^− 17^). This is probably due to the addition of specific signals in the genomic environment of the gene (further than 150 bp from the gene boundaries), such as the promoter, enhancers/silencers, etc. that are taken into account in the program prediction models. At the exon level, the effect of the flanking sequences is not the same for the different programs. For example, the sensitivity of Augustus (p-value = 4.06 × 10^− 4^), Genscan (p-value = 1.59 × 10^− 8^) and GeneID (p-value = 2.98 × 10^− 2^) is highest when the input sequence has 150 bp flanking regions and significantly decreases when 2Kb flanking nucleotides are added, while for GlimmerHMM (p-value = 0.54) and Snap (p-value = 0.62) no significant difference is observed. Similar results are observed in terms of specificity. At the protein level, for all five programs, the sequence identity compared to the benchmark protein sequence decreases as the length of the flanking sequences increases.

For Augustus, Genscan and GeneID, the addition of the flanking sequences also reduces the number of proteins perfectly predicted (100% identity). This is especially true for Genscan, where we observe a loss of more than 24% of perfectly predicted proteins between 150 bp and 2Kb. On the other hand, for GlimmerHMM and Snap, the number of perfectly predicted proteins increases, especially when 2-4Kb flanking DNA is provided.

Since the greatest effect of adding upstream/downstream flanking sequences was generally observed for a length of 2Kb, the remaining analyses described in this work are all based on the gene sequences with 2Kb upstream/downstream flanking regions.

**Fig. 8:**
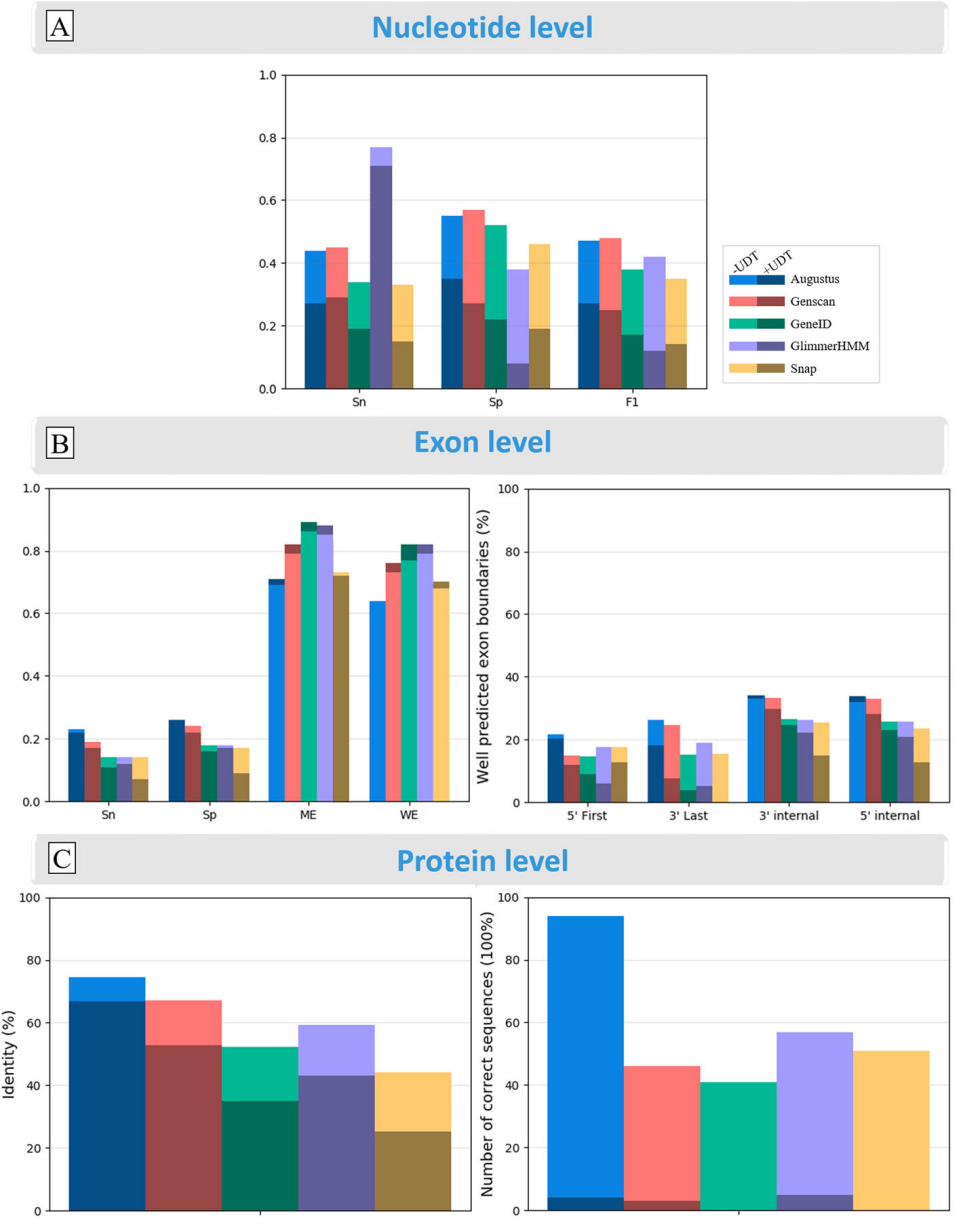
Effect of undetermined sequence regions (UDT) on prediction performance of the five gene prediction programs, using Confirmed benchmark sequences from Metazoa, where 542 sequences have no undetermined regions (−UDT: light colors) and 133 sequences have undetermined regions (+UDT: dark colors). **a** sensitivity and specificity of nucleotide prediction. **b** sensitivity and specificity of exon prediction **c** accuracy of protein sequence prediction (% identity) and number of proteins correctly predicted with 100% identity. Sn = sensitivity; Sp = specificity; F1 = F1 score; ME = Missing Exons; WE = Wrong Exons; 5′ First = percentage of correctly predicted 5′ boundaries of first exons only; 3′ Last = percentage of correctly predicted 3′ boundaries of last exons; 3′ and 5′ internal are the percentage of correctly predicted 3′ and 5′ internal exon boundaries. %Identity indicates the sequence identity observed between the predicted proteins and the Confirmed benchmark sequences

Next, we studied the relative robustness of the programs to the presence of UDT regions in the genomic sequences, generally due to genome sequencing errors or assembly gaps. This test was limited to the Confirmed sequences from the metazoan clade, since the sequences with UDT regions were almost exclusively found in this clade. Of the 675 metazoan sequences, 133 were found to have UDT regions. We therefore compared the 542 Confirmed sequences without UDT (−UDT) regions with the 133 Confirmed sequences with UDT regions (+UDT). Figure 8 and Additional file 1: Table S9 show the average scores obtained for these two sequence sets, at the nucleotide, exon and protein levels. As might be expected, a reduction in sensitivity and specificity was observed at the nucleotide and exon levels for almost all programs (except exon level specificity and 5′/3′ internal exon boundaries of Augustus) for the +UDT sequences, and at the protein level, very few +UDT proteins are predicted with 100% accuracy. Overall, Augustus and Genscan perform better, although GlimmerHMM predicts the highest number of proteins with 100% accuracy for the +UDT sequences.

Since the UDT regions affected the programs to different extents, the analyses described in the following sections are all based on the set of 756 Confirmed sequences that have no UDT regions.

Finally, we investigated how the GC content of the genes influences the gene finders (Additional file 1: Fig. S6). As might be expected, genes with high GC content are predicted better than genes with high AT content. The GC content of the genome is more difficult to test independently of the other factors, but could contribute to the species-dependent differences observed, shown in Fig. 12.

#### Factors associated with the gene structure

**Fig. 9:**
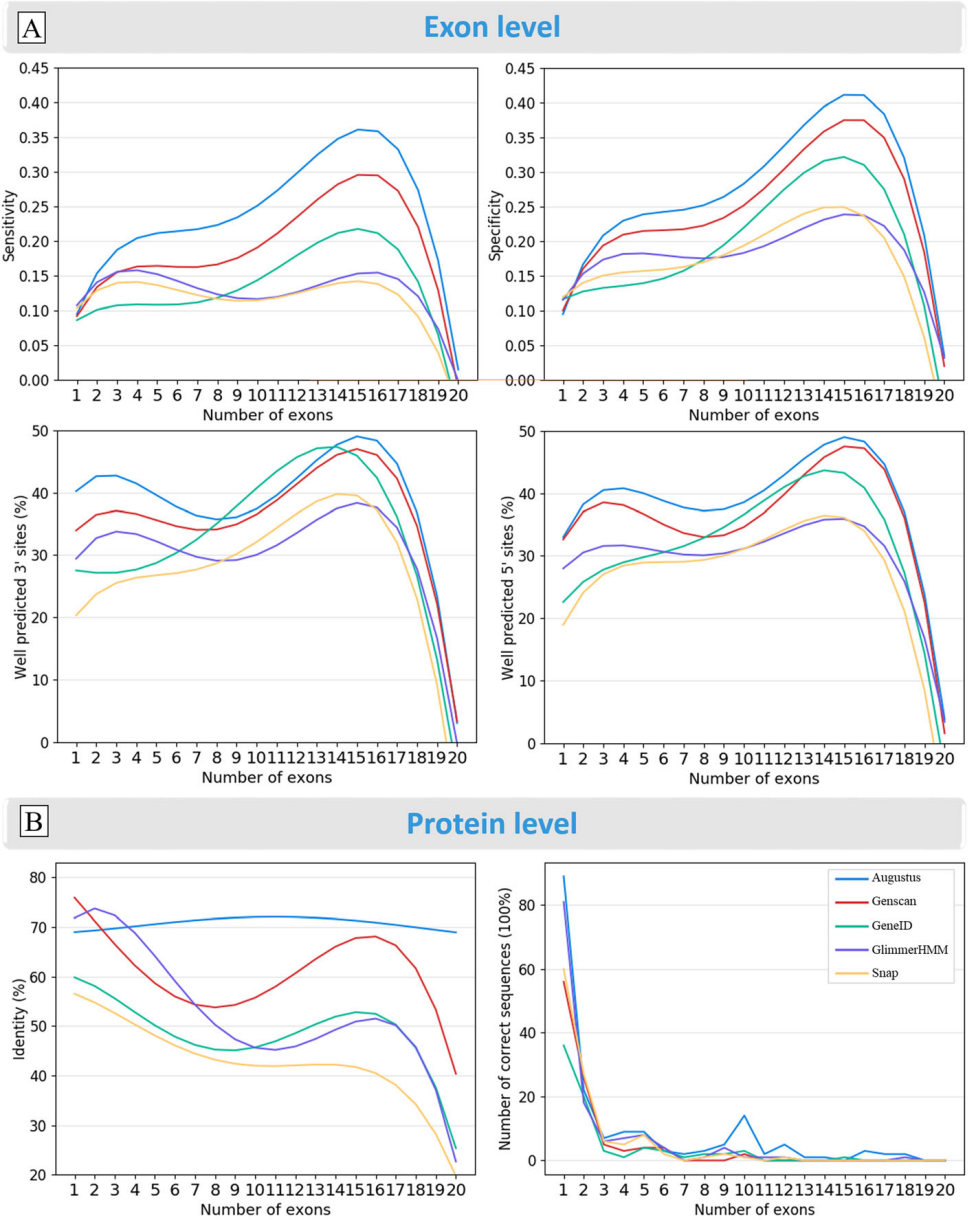
Effect of exon map complexity on prediction quality at the **a** exon and **b** protein levels. A 4th degree polynomial curve fitting was used to represent the results more clearly. Sequences with 21–24 exons were not included, due to the low number of sequences in the benchmark with these exon counts. 3′ and 5′ are the proportion of correctly predicted 3′ and 5′ internal exon boundaries respectively. %Identity indicates the sequence identity observed between the predicted proteins and the Confirmed benchmark sequences

We first evaluated the effect of the Exon Map Complexity (EMC), represented by the number of exons in the Confirmed benchmark tests (Additional file 1: Fig. S7). Figure 9 shows the quality scores at the exon and protein levels, for sequences with the number of exons ranging from 1 to 20. Overall, we observed a tendency for the five programs to achieve better sensitivity and specificity for the genes with more exons. This may be because most of these more complex sequences are from well-studied vertebrate genomes. For very complex exon maps (≥20 exons), all the programs seem to perform less well, although this may be an artifact due to the small number of these sequences in the benchmark (Additional file 1: Fig. S7A). For single exon genes, all the programs tend to perform worse, although the 3′ internal exon boundary of the cDNA is predicted better than the 5′ internal exon boundary. Similarly, the 3′ internal exon boundaries are generally predicted better than the 5′ internal exon boundaries by all the programs, for genes with a small number of exons. At the protein level, Augustus and GlimmerHMM achieve higher sequence identity for genes with ≤7 exons, while Augustus and Genscan are more accurate for genes with more exons. Most of the perfectly predicted proteins (with 100% sequence identity) have less than 3 exons.

**Fig. 10:**
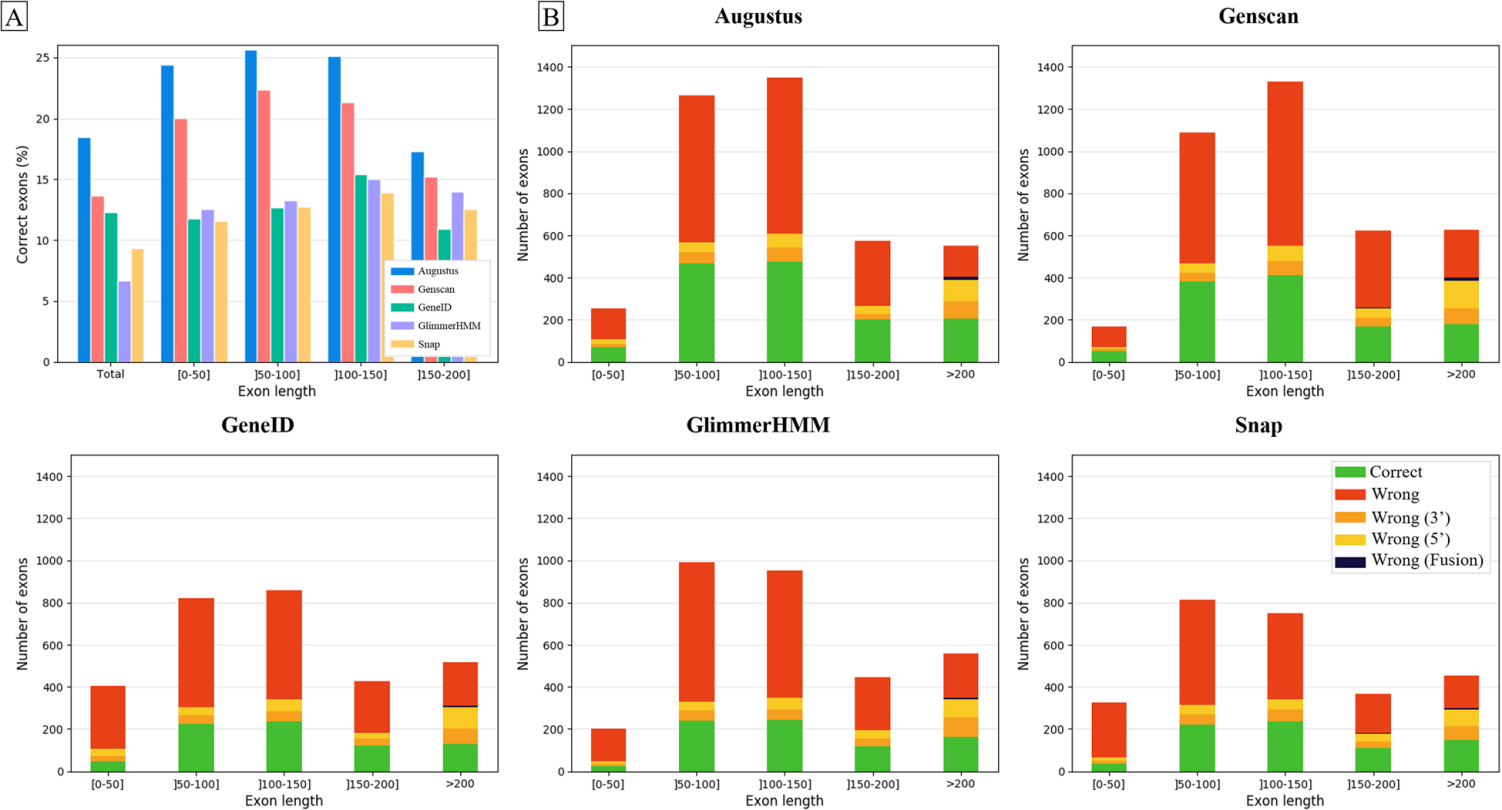
Effect of exon length on exon prediction quality. **a** Proportion of benchmark exons correctly predicted depending on the exon length. **b** Number of exons predicted correctly, with one of the 5′ or 3′ exon boundaries correct, or with both boundaries wrongly predicted, for each of the five programs

We then assessed the effect of exon lengths on the prediction quality of the five programs, using the 756 Confirmed sequences without UDT regions. Figure 10a and Additional file 1: Table S10A show the proportion of Correct exons (both 5′ and 3′ exon boundaries correctly predicted) depending on the exon length. The short exons (< 50 nucleotides) are generally the least accurate, with the best program, Augustus, achieving only 18% Correct short exons. Medium length exons (50–200 nucleotides) are predicted better than longer exons (> 200 nucleotides) for Augustus and Genscan.

To further investigate the exon prediction, each exon predicted by a gene prediction program was classified as ‘Correct’ if both exon boundaries were correctly predicted, ‘Wrong (5’)’ or ‘Wrong (3′)’ if the 5′ or 3′ exon boundary was badly predicted respectively, and ‘Wrong’ if both boundaries were badly predicted. In some cases, the predicted exon has good 5′ and 3′ exon boundaries, however they correspond to 2 different benchmark exons, so these exons are classed as ‘Wrong (Fusion)’. Figure 10b and Additional file 1: Table S10B show the number of Correct, Wrong, Wrong (5′), Wrong (3′) and Wrong (Fusion) exons, according to the exon lengths. Overall, there are more ‘Wrong’ exons than ‘Correct’ exons for all exon lengths and for all the programs. Interestingly, the number of predicted exons with only one boundary correctly predicted, i.e. Wrong (5′) or Wrong (3′), is small for all exon lengths, except for exons with > 200 nucleotides.

#### Factors associated with the protein product

In this section, prediction accuracy is measured at the protein level and is estimated by the percent sequence identity of the predicted protein compared to the benchmark protein.

**Fig. 11:**
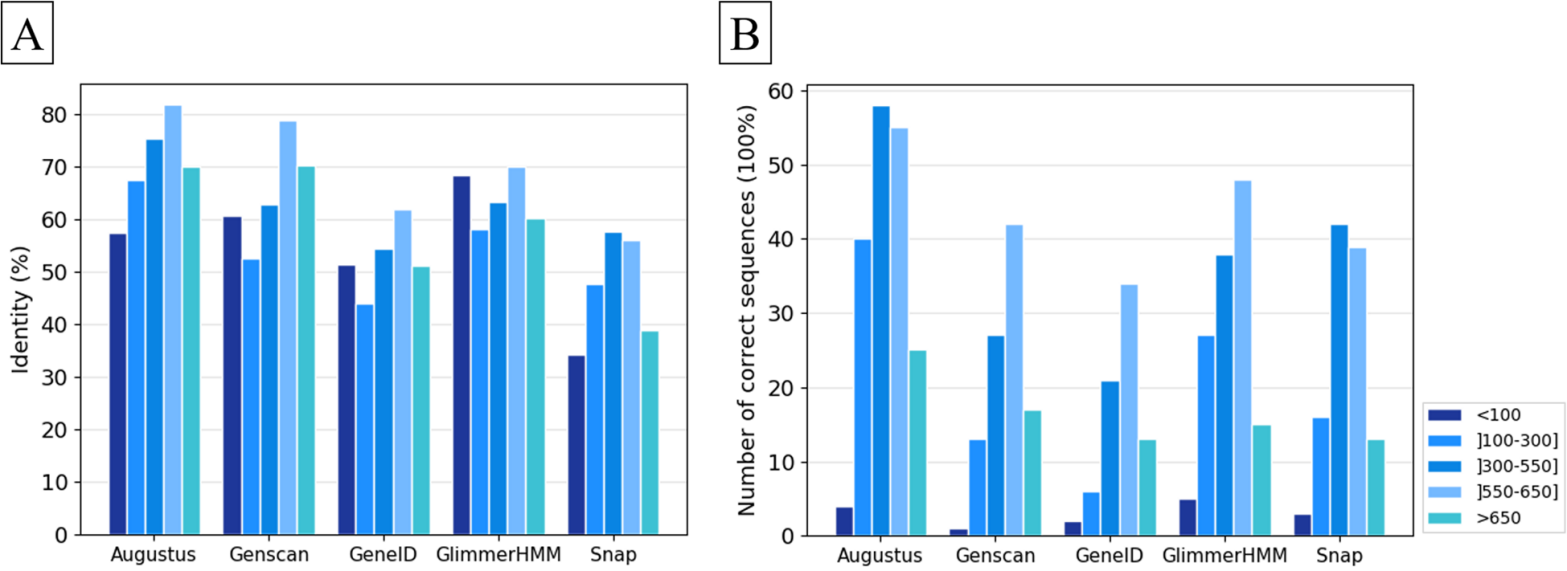
Effect of protein length on prediction accuracy: **a** average percent identity between the predicted and the benchmark protein sequences, **b** number of proteins perfectly predicted with 100% sequence identity

First, we investigated the effect of protein length on protein prediction quality. We divided the 756 Confirmed sequences without UDT regions into five groups, with different protein lengths ranging from 50 to 1000 amino acids (Additional file 1: Fig. S8). Note that the very large proteins (> 1000 amino acids) in the benchmark are all classified as Unconfirmed and are therefore not included in this study. Figure 11 and Additional file 1: Table S11 show the mean accuracies obtained by the five programs for the different length proteins. The prediction accuracy generally decreases for shorter proteins and for protein lengths > 650 amino acids. For proteins with < 100 amino acids, GlimmerHMM achieves the best results with 68% sequence identity and five (25%) perfectly predicted proteins (100% identity), while Augustus obtains only 57% sequence identity and four perfectly predicted proteins.

**Fig. 12:**
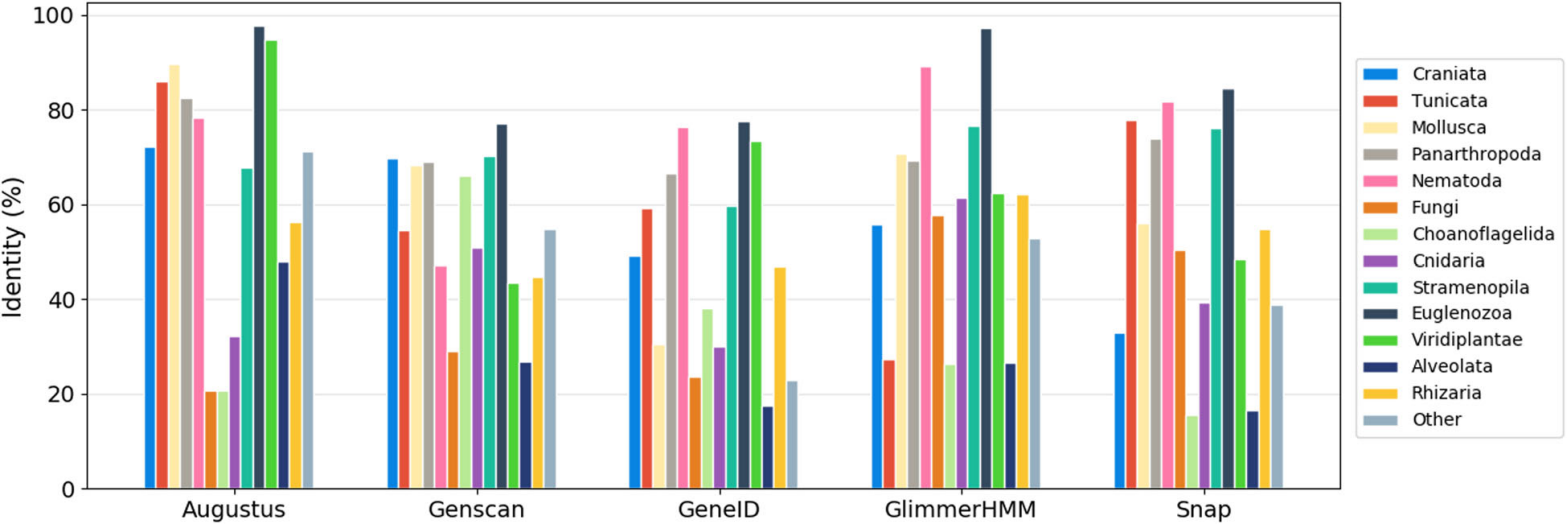
Prediction performance for sequences from different clades. The ‘Other’ group contains the Apusozoa, Cryptophyta, Diplomonadida, Haptophyceae, Heterolobosea, Parabasalia clades, as well as Placozoa, Annelida and urchin. % Identity indicates the average percent identity between the predicted and the benchmark protein sequences

We then studied the phylogenetic origin of the proteins and the availability of suitable species models in the different programs. Figure 12 and Additional file 1: Table S12 show the performance of the five gene prediction programs for the sequences in the different clades in G3PO. The accuracy of each program is highly variable between the different clades, probably due to the availability of suitable prediction models for some species. For the sequences in the Craniata clade, Augustus and Genscan achieve the highest accuracy (72 and 70% respectively), while Snap has the lowest accuracy (33%). In contrast, Augustus obtains lower accuracy (21%) for Fungi proteins, compared to the highest accuracy obtained by GlimmerHMM (58%). The proteins in the Euglenoezoa clade are predicted with the highest accuracy by all the programs, although this might be explained by their low EMC. Choanoflagellida and Cnidaria proteins are the least well predicted (except for Genscan), but these clade contain only a few sequences (5 and 6 sequences respectively) and this result remains to be confirmed.

#### Effect of protein sequence errors

Finally, we investigated the performance of the prediction programs for the 904 Unconfirmed sequences, where potential sequence errors were observed in the benchmark sequences. As mentioned above, the G3PO benchmark sequences were extracted from the Uniprot database, which means that many of the proteins are not supported by experimental evidence. In this test, we wanted to estimate the prediction accuracy of the five gene prediction programs for the Unconfirmed benchmark sequences. Since the Unconfirmed sequences could not be used as a ground truth, here we measured prediction accuracy based on a closely related Confirmed sequence (see Methods). Table 2 shows the prediction accuracies achieved by each program for the sets of Confirmed and Unconfirmed sequences. As might be expected, the Unconfirmed sequences are predicted with lower accuracy than the Confirmed sequences by all five programs. Augustus and Genscan achieved the highest accuracy (56, 50% respectively) for the Unconfirmed sequences. For comparison purposes, we also calculated the accuracy scores for the Unconfirmed benchmark proteins. The benchmark proteins had higher accuracy (76%) than any of the methods tested here, implying that the more complex pipelines used to curate proteins in Uniprot can effectively improve the results of ab initio methods.

**Table 2:** Effect of protein sequence quality measured at the protein level. %Identity indicates the average sequence identity observed between the predicted and benchmark protein sequences for the test sets of Confirmed and Unconfirmed proteins

	Confirmed proteins (%Identity)	Unconfirmed proteins (%Identity)
Augustus	74.44	56.22
Genscan	67.13	49.86
GeneID	52.26	38.52
GlimmerHMM	59.36	45.60
Snap	44.20	41.70

## Discussion

Several recent reviews [3, 22, 23] have highlighted the fact that automated genome annotation strategies still have difficulty correctly identifying protein-coding genes. This failure might be explained by the quality of the draft genome assemblies, the complexity of eukaryotic exon maps, high levels of genetic sequence divergence or deviations from canonical genetic characteristics [36]. Consequently, it is essential to benchmark the existing different gene prediction strategies to assess their reliability, to identify the most promising approaches, but also to limit the spread of errors in protein databases [37]. An ideal benchmark for gene prediction programs should include proteins encoded by real genomic sequences. Unfortunately, most of the protein sequences in the public databases have not been verified by experimental means, with the exception of the manually annotated Swiss-Prot sequences (representing only 0.3% of UniProt), and contain many sequence annotation errors. It is therefore dangerous to use them to estimate the accuracy of the prediction programs.

G3PO is a new gene prediction benchmark containing 1793 orthologous sequences from 20 different protein families, and designed to be as representative as possible of the living world. It includes sequences from phylogenetically diverse organisms, with a wide range of different genomic and protein characteristics, from simple single exon genes to very long and complex genes with over 20 exons. The quality of the protein sequences in the benchmark was ensured by excluding sequences containing potential annotation errors, including deletions, insertions and mismatched segments. We also characterized the test sets in the benchmark using different features at the genome, gene structure and protein levels. This in-depth characterization allowed us to investigate the impact of these features on gene prediction accuracy.

One of the main limitations of the benchmark concerns the fact that the protein sequences were extracted from the Uniprot database, where a ‘canonical’ protein isoform is defined based on cross-species conservation and the conservation of protein structure and function. Consequently, programs that predicted more minor isoforms created by alternative splicing events were penalized in our evaluations. Unfortunately, there is currently no ideal solution to this. In the future, gene prediction programs will need to evolve to predict all isoforms for a gene. Another limitation of the benchmark concerns the evaluation of the gene prediction results with respect to a single benchmark sequence. It is possible that the flanking regions used in some tests covered more than one gene, and that some programs successfully predicted one or more exons from these neighboring genes in addition to the reference gene.

The ab initio gene prediction programs included in the benchmark study are based on statistical models that are trained using known proteins and genes, and typically perform well at predicting conserved or well-studied genes [33, 38]. However, ab initio prediction accuracy has been previously shown to decrease in some special cases, such as small proteins [39], organism-specific genes or other unusual genes [40–42]. Our goal was therefore to identify the strengths and weaknesses of the programs, but also to highlight genomic and protein characteristics that could be incorporated to improve the prediction models.

In terms of overall quality, the gene prediction programs were generally ranked in agreement with previous findings, with Augustus and Genscan achieving the best overall accuracy scores. However, it should be noted that Augustus is also the most computationally expensive method, taking over 1 h to process the 87 Mb corresponding to the 1793 benchmark sequences, compared to the fastest program, GeneID, which required only 4 min.

We then performed a more in-depth study of the different factors affecting prediction accuracy. At the genome level, an increase in accuracy was generally observed when at least 2Kb flanking regions were added, reflecting the fact that all the programs try to model in vivo gene translation systems to some extent by taking into account the different regulatory signals found within and outside the gene [43]. In contrast, undetermined regions in the gene sequences had a negative effect on the accuracy of all the prediction programs, even when they occur outside the coding exons of the genes. Since undetermined or ambiguous regions are likely to occur more often in low coverage genomes, this is an important issue that needs to be addressed by the developers of gene prediction software.

At the gene structure level, we found that the number of exons affects the accuracy of all the programs and that gene prediction is generally more difficult for complex exon maps, as might be expected. Concerning the effect of exon length, the programs appear to be optimized for intermediate length exons (50–200 nucleotides), since none of the programs was able to reliably predict exons that were shorter (< 50 nucleotides) or longer (> 200 nucleotides). Protein length had a similar effect to that observed for exon length, since the programs seem to be optimized for intermediate length proteins (300–650 amino acids). This result confirms previous findings that smaller proteins (less than 100 amino acids) are often missed in genome annotations [39], although we also demonstrated that long proteins are also more likely to be badly predicted. Finally, the phylogenetic origin of the benchmark sequences had a large effect on prediction accuracy, with different programs producing the best results depending on the specific species. The two best scoring programs, Augustus and Genscan use different strategies, since Augustus includes > 100 different species models, while Genscan has only three models.

**Fig. 13:**
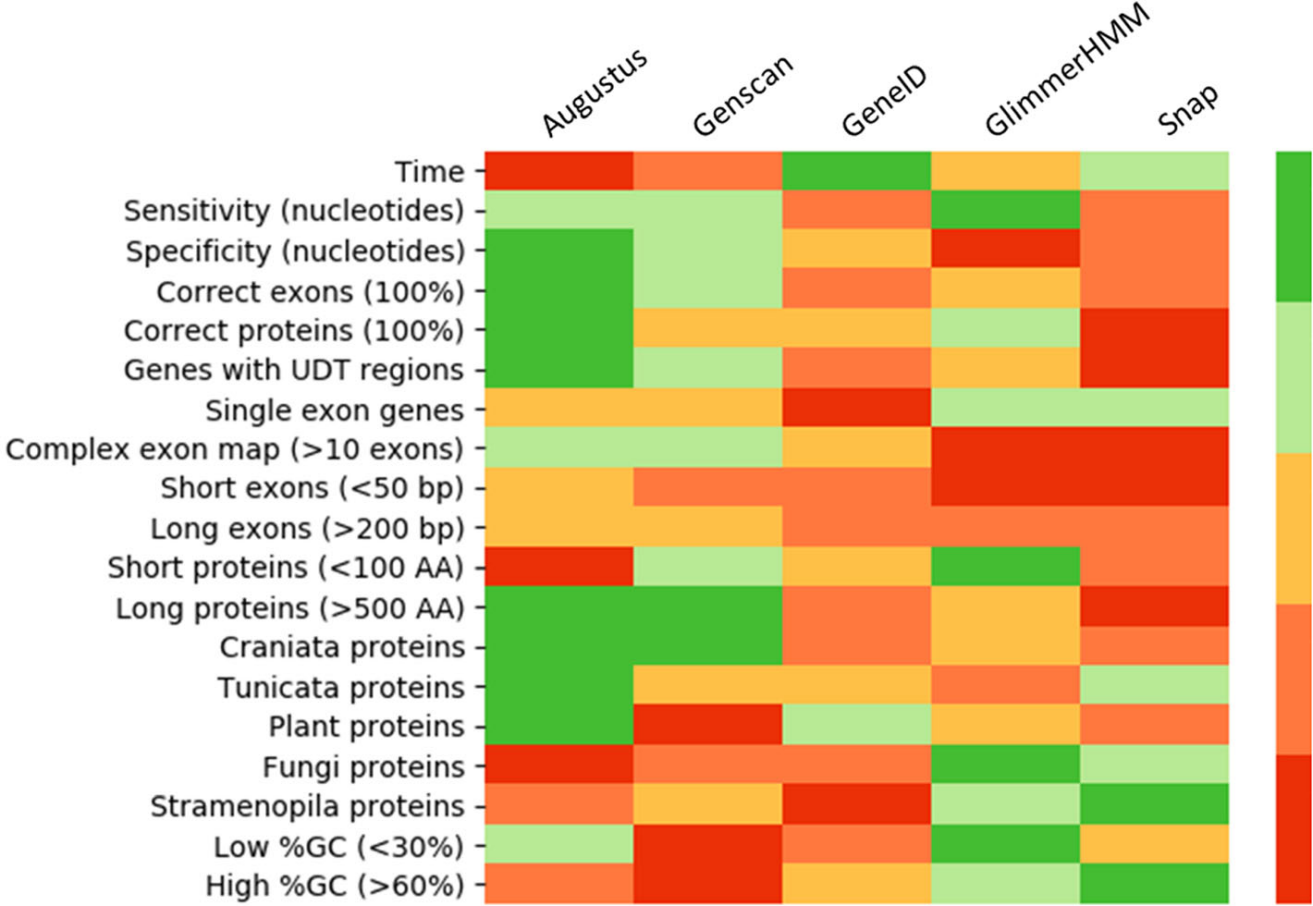
Strengths and weaknesses of the gene prediction programs evaluated in this study. Heatmap colors are: dark green = best program, light green = 2nd best program, yellow = 3rd best program, orange = 4th best program, red = 5th best program

Each of the analyses performed here highlights different strengths or weaknesses of the prediction programs, as summarized in the heat map shown in Fig. 13. The in-depth characterization of the benchmark sequences and the detailed information extracted from the analyses provide essential elements that could be used to improve model training and therefore gene prediction. It may be interesting to further analyze the weaknesses identified, including small proteins, very long proteins, proteins coded by a large number of exons, proteins from non-model organisms, etc.

Finally, the Unconfirmed sequences identified in this study represent a goldmine for the identification of atypical gene features, for example atypical regulatory signals or splice sites, that are not fully taken into account in the current prediction models. More than 50% of the original reference protein sequences extracted from public databases were found to contain at least one error. They therefore represent very challenging test cases that were not resolved by the combined ab initio and similarity-based curation processes used to annotate these proteins. We accurately located the errors within these badly predicted sequences and classified them into 9 groups. Here, we performed a preliminary analysis using the erroneous sequences that confirmed our idea that all the prediction programs are less accurate for these proteins. A more comprehensive analysis of these proteins will be published elsewhere.

## Conclusions

The complexity of the genome annotation process and the recent activity in the field mean that it is timely to perform an extensive benchmark study of the main computational methods employed, in order to obtain a more detailed knowledge of their advantages and disadvantages in different situations. Currently, most of the programs used for gene prediction are based on statistical approaches and perform relatively well in intermediate cases. However, they have difficulty identifying more extreme cases, such as very short or very long proteins, complex exon maps, or genes from less well studied species. Recently, artificial intelligence approaches have been applied to some specific tasks, for example DeepSplice [44] or SpliceAI [45] for the prediction of splice sites. The further development of these approaches should contribute to production of high quality gene predictions that can be leveraged downstream to improve functional annotations, evolutionary studies, prediction of disease genes, etc.

## Methods

### Benchmark test sets

**Fig. 14:**
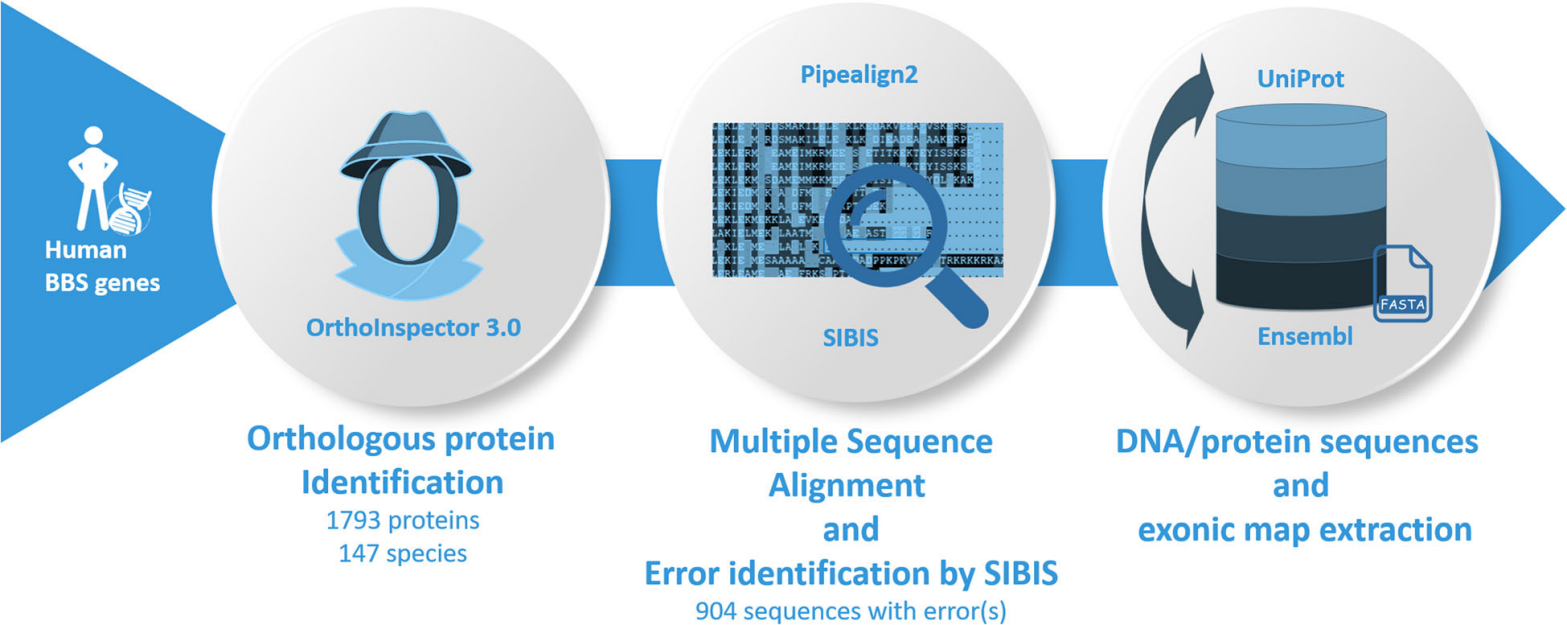
Schematic view of the pipeline used to construct the benchmark

To construct a benchmark set of eukaryotic genes, we selected the 20 human Bardet-Biedl Syndrome (BBS) proteins (Additional file 1: Table S2). Based on this initial gene set, we extended the test sets using the pipeline shown in Fig. 14 and described in detail below.

(i) For each of the 20 human proteins, orthologous proteins were identified in 147 eukaryotic organisms (Additional file 1: Table S1) using OrthoInspector version 3.0 [46], which was built using proteins from the Uniprot Reference Proteomes database [34] (Release 2016_11). For each species, we selected one ortholog sharing the highest percent identity with the human sequence. This resulted in a total of 1793 protein sequences, of which 65 (3.6%) were found in the curated Swissprot database. The number of proteins in each BBS family is provided in Additional file 1: Table S2. BBS 6,10,11,12,15, 16 and 18 are specific to Metazoa (with some exceptions), and therefore contain fewer sequences than the other families.
(ii) Since the reference protein sequences extracted from the Uniprot database may contain errors, we identified potentially unreliable sequences based on multiple sequence alignments (MSA). MSAs were constructed for each protein family using the Pipealign2 tool (http://www.lbgi.fr/pipealign) and manually refined to identify and correct misaligned regions. The SIBIS (version 1.0) program [47] using a Bayesian framework combined with Dirichlet mixture models and visual inspection, was used to identify inconsistent sequence segments. These segments might indicate that different isoforms are defined as the canonical sequence for different organisms, or they might indicate a badly predicted protein (Additional file 1: Fig. S3). SIBIS classifies the potential sequence errors into 9 categories: N-terminal deletion, N-terminal extension, N-terminal mismatched segment, C-terminal deletion, C-terminal extension, C-terminal mismatched segment, internal deletion, internal insertion and internal mismatched segment. Of the 1793 protein sequences identified in step (i), 889 proteins had no errors (called “Confirmed”) and 904 proteins had at least one potential error (called “Unconfirmed”). At this stage, the BBS14 protein was excluded from the benchmark because the MSA contained too many misalignments.
(iii) For each orthologous protein, the genomic sequence was extracted from the Ensembl database [35]. Genomic sequences were extracted with the ‘soft mask’ option, i.e. repeated or low complexity regions are replaced by lower case nucleotides. These are generally ignored by gene prediction programs. We also found regions with ‘n’ characters, which are used to indicate undetermined or ambiguous nucleotides (IUPAC nomenclature) probably caused by genome sequencing errors or assembly gaps. A sequence segment with a run of n characters was defined as an undetermined (UDT) region. Additional file 1: Table S5 summarizes the general statistics of these 283 sequences with UDT regions. Finally, we identified the Ensembl transcript corresponding to the Uniprot protein sequence, (generally the ‘canonical transcript’ from APPRIS [48]) in order to construct the exon map by extracting the positions of all exons/introns, including the 5′/3′ untranslated regions when available.
(iv) For the baseline tests, we included flanking sequences of length 150 bases upstream and downstream of the gene. To make the benchmark set more challenging, we also extracted genomic sequences corresponding to 2Kb, 4Kb, 6Kb, 8Kb, 10Kb upstream and downstream of the gene sequence.

### Gene prediction methods

The programs tested are listed in Table 1 with the main features, including the HMM model used to differentiate intron/exon regions, and the specific signal sensors used to detect the presence of functional sites. Transcriptional signal sensors include the initiator or cap signal located at the transcriptional start site and the upstream TATA box promoter signal, as well as the polyadenylation signal (a consensus AATAAA hexamer) located downstream of the coding region and the 3′ UTR. Translational signals include the “Kozak sequence” located immediately upstream of the start codon [49]. For higher eukaryotes, splice site signals are also incorporated, including donor and acceptor sites (GT-AG on the intron sequence) and the branch point [yUnAy**]** [50] (underlined A is the branch point at position zero and y represents pyrimidines, n represents any nucleotide) located 20–50 bp upstream of the AG acceptor.

The command lines used to run the programs are:

augustus --species=<species> --softmasking=1 --gff3=off <sequence.fasta>

genscan <species> <sequence.fasta>

geneid -A -P <species> <sequence.fasta>

glimmerhmm <sequence.fasta> -d <species> -g

snap -gff -quiet -lcmask <species> <sequence.fasta> --a protein.fasta

where <species> indicates the species model used and < sequence.fasta> contains the input genomic sequence.

All programs were run on an Intel(R) Xeon(R) CPU E5–2695 v2 @ 2.40Ghz, 12 cores, with 256 Go RAM. Each prediction program was run with the default settings, except for the species model to be used. As the benchmark contains sequences from a wide range of species, we selected the most pertinent training model for each target species, based on the taxonomic proximity between the target and model species. For each program, we compared the taxonomy of the target species with the taxonomy for each model species available, where taxonomies were obtained from the NCBI Taxonomy database (https://www.ncbi.nlm.nih.gov/taxonomy). We then selected the model species that was closest to the target in the taxonomic tree.

### Evaluation metrics

The performance of the gene prediction programs is based on the measures used in [29], calculated at three different levels: nucleotides, exons and complete proteins. The significance of pairwise comparisons of the evaluation metrics was evaluated using the paired t-test.

At the nucleotide level, we measure the accuracy of a gene prediction on a benchmark sequence by comparing the predicted state (exon or intron) with the true state for each nucleotide along the benchmark sequence. Nucleotides correctly predicted to be in either an exon or an intron are considered to be True Positives (TP) or True Negatives (TN) respectively. Conversely, nucleotides incorrectly predicted to be in exons or introns are considered to be False Positives (FP) or False Negatives (FN) respectively. We then calculated different performance statistics, defined below.

Sensitivity measures the proportion of benchmark nucleotides that are correctly predicted:

$$ Sn=\frac{TP}{TP+ FN.} $$

The specificity measure that is most widely used in the context of gene prediction is the proportion of nucleotides predicted in exons that are actually in exons:

$$ Sp=\frac{TP}{TP+ FP} $$

The F1 score represents the harmonic mean of the sensitivity and specificity values:

$$ F1=2\ast \frac{Sp\ast Sn}{Sp+ Sn} $$

At the exon structure level, we measure the accuracy of the predictions by comparing predicted and true exons along the benchmark gene sequence. An exon is considered correctly predicted (TP), when it is an exact match to the benchmark exon, i.e. when the 5′ and 3′ exon boundaries are identical. All other predicted exons are then considered FP. Sensitivity and specificity are then defined as before.

Since the definition of TP and TN exons above is strict, we also calculated two additional measures similar to those defined in [29] (Additional file 1: Fig. S9). First, true exons with or without overlap to predicted exons are considered to be Missing Exons (ME) and the MEScore is defined as:

$$ MEScore=\frac{ME}{Total\ number\ of\ true\ exons} $$

Second, predicted exons with or without overlap to true exons are considered Wrong Exons (WE). The WEScore is defined as:

$$ WEScore=\frac{WE}{Total\ number\ of\ predicted\ exons\ } $$

We also determined the proportion of correctly predicted 5′ and 3′ exon boundaries, as follows:

$$ 5^{\prime }=\frac{\mathrm{number}\ \mathrm{of}\ \mathrm{true}\ {5}^{\prime }\ \mathrm{exon}\ \mathrm{boundaries}\ \mathrm{correct}\mathrm{ly}\ \mathrm{predicted}\ast 100}{\mathrm{number}\ \mathrm{of}\ \mathrm{correct}\ \mathrm{predicted}\ \mathrm{exons}+\mathrm{number}\ \mathrm{of}\ \mathrm{wrong}\ \mathrm{exons}} $$

$$ 3^{\prime }=\frac{\mathrm{number}\ \mathrm{of}\ \mathrm{true}\ {3}^{\prime }\ \mathrm{exon}\ \mathrm{boundaries}\ \mathrm{correct}\mathrm{ly}\ \mathrm{predicted}\ast 100}{\mathrm{number}\ \mathrm{of}\ \mathrm{correct}\ \mathrm{predicted}\ \mathrm{exons}+\mathrm{number}\ \mathrm{of}\ \mathrm{wrong}\ \mathrm{exons}} $$

At the protein level, we measure the accuracy of the protein products predicted by a program. Since a program may predict more than one transcript for a given gene sequence in the benchmark, we calculate the percent identity between the benchmark protein and all predicted proteins and the predicted protein with the highest percent identity score is selected. To calculate the percent identity score between the benchmark protein and the predicted protein, we construct a pairwise alignment using the MAFFT software (version 7.307) [51] and the percent identity is then defined as:

$$ \% Identity=\frac{Number\ of\ identical\ amino\ acids\ast 100}{Length\ of\ benchmark\ protein} $$

### Evaluation metric for unconfirmed benchmark proteins

Since the Unconfirmed proteins in the benchmark are badly predicted and have at least one identified sequence error, the %Identity score defined above for the Confirmed sequences cannot be used. Instead, we compare the protein sequences predicted by the programs with the most closely related Confirmed sequence found in the corresponding MSA. Thus, for a given Unconfirmed sequence, *E*, we calculated the sequence identity between *E* (excluding the sequence segments with predicted errors) and all the orthologous sequences in the corresponding MSA. If a Confirmed orthologous sequence, *V*, was found that shared ≥50% identity with *E*, then the sequence *V* was used as the reference protein to evaluate the program prediction accuracy.

As before, a pairwise alignment between the prediction protein and sequence *V* was constructed using MAFFT and the %Identity score was calculated. Finally, the accuracy score was normalized by the sequence identity shared between the *E* and *V* benchmark sequences.

$$ Accuracy=\frac{\% Identity\left(P,V\right)\ast 100}{\% Identity\left(E,V\right)} $$

## Supplementary information

**Additional file 1: Tables S1–11**, **Figures S1–9**.

## Data Availability

The DNA and protein sequences used in the G3PO benchmark, the scripts used to produce the results and the outputs of the gene prediction programs are available at http://git.lbgi.fr/scalzitti/Benchmark_study.

## Abbreviations

AA: Amino acid
BBS: Bardet-Biedl syndrome
Bp: Base pair
DNA: Deoxyribonucleic acid
EMC: Exon map complexity
F1: F1 score
FN: False negative
FP: False positive
HMM: Hidden Markov Model
Kb: Kilobase
ME: Missing exon
MSA: Multiple Sequence Alignment
RNA: Ribonucleic acid
Sn: Sensitivity
Sp: Specificity
TN: True negative
TP: True positive
UDT: Undetermined region
UTR: Untranslated region
WE: Wrong exon
