# Supplementary material for: A benchmark study of ab initio gene prediction methods in diverse eukaryotic organisms
Source: BMC Genomics. 2020 Apr 9;21:293. doi: 10.1186/s12864-020-6707-9 (PMC7147072; doi:10.1186/s12864-020-6707-9)
Supplement: Supplementary file 1 — Additional file 1: Tables S1–11, Figures S1–9. [file 12864_2020_6707_MOESM1_ESM.pdf]

UNIPROT Species identifier	*Species name*	UNIPROT Species identifier	*Species name*
ACYPI	*Acyrthosiphon pisum*	MUSPF	*Mustela putorius furo*
AEDAE	*Aedes aegypti*	MYOLU	*Myotis lucifugus*
AILME	*Ailuropoda melanoleuca*	NAEGR	*Naegleria gruberi*
ALLMA	*Allomyces macrogynus ATCC 38327*	NASVI	*Nasonia vitripennis*
ANOCA	*Anolis carolinensis*	NEMVE	*Nematostella vectensis*
ANODA	*Anopheles darlingi*	NOMLE	*Nomascus leucogenys*
ANOGA	*Anopheles gambiae*	NOSCE	*Nosema ceranae (strain BRL01)*
ASTMX	*Astyanax mexicanus*	ONCVO	*Onchocerca volvulus*
ATTCE	*Atta cephalotes*	ORENI	*Oreochromis niloticus*
AURAN	*Aureococcus anophagefferens*	ORNAN	*Ornithorhynchus anatinus*
BATDJ	*Batrachochytrium dendrobatidis (strain JAM81 / FGSC 10211)*	ORYLA	*Oryzias latipes*
BETVU	*Beta vulgaris subsp. vulgaris*	OTOGA	*Otolemur garnettii*
BODSA	*Bodo saltans*	PANTR	*Pan troglodytes*
BOMMO	*Bombyx mori*	PAPAN	*Papio anubis*
BOVIN	*Bos taurus*	PARTE	*Paramecium tetraurelia*
BRUMA	*Brugia malayi*	PEDHC	*Pediculus humanus subsp. corporis*
CAEBE	*Caenorhabditis brenneri*	PELSI	*Pelodiscus sinensis*
CAEBR	*Caenorhabditis briggsae*	PERM5	*Perkinsus marinus (strain ATCC 50983 / TXsc)*
CAEEL	*Caenorhabditis elegans*	PHAAN	*Phaseolus angularis*
CAERE	*Caenorhabditis remanei*	PHYIT	*Phytophthora infestans (strain T30-4)*
CALJA	*Callithrix jacchus*	PHYNI	*Phytophthora nicotianae*
CANLF	*Canis lupus familiaris*	PHYPN	*Phytophthora parasitica (strain INRA-310)*
CAVPO	*Cavia porcellus*	PHYPR	*Phytophthora parasitica P1569/ Phytophthora parasitica P10297*
CHICK	*Gallus gallus*	PHYRM	*Phytophthora ramorum*
CHLRE	*Chlamydomonas reinhardtii*	PHYSP	*Phytophthora sojae (strain P6497)*
CHLSB	*Chlorocebus sabaeus*	PIG	*Sus scrofa*
CIOIN	*Ciona intestinalis*	PLABS	*Plasmodiophora brassicae*
CIOSA	*Ciona savignyi*	POEFO	*Poecilia formosa*
CRAGI	*Crassostrea gigas*	PONAB	*Pongo abelii*
CULQU	*Culex quinquefasciatus*	PRIPA	*Pristionchus pacificus*
DANRE	*Danio rerio*	PSEPJ	*Pseudocohnilembus persalinus*
DAPPU	*Daphnia pulex*	PYTUL	*Pythium ultimum DAOM BR144*
DENPD	*Dendroctonus ponderosae*	RABIT	*Oryctolagus cuniculus*
DROAN	*Drosophila ananassae*	RAT	*Rattus norvegicus*
DROGR	*Drosophila grimshawi*	RETFI	*Reticulomyxa filosa*
DROME	*Drosophila melanogaster*	RHOPR	*Rhodnius prolixus*
DROMO	*Drosophila mojavensis*	SALR5	*Salpingoeca rosetta (strain ATCC 50818 / BSB-021)*
DROPE	*Drosophila persimilis*	SAPPC	*Saprolegnia parasitica (strain CBS 223.65)*
DROPS	*Drosophila pseudoobscura pseudoobscura*	SARHA	*Sarcophilus harrisii*

DROSE	*Drosophila sechellia*	SARSC	*Sarcoptes scabiei*
DROSI	*Drosophila simulans*	SCHMA	*Schistosoma mansoni*
DROVI	*Drosophila virilis*	SELML	*Selaginella moellendorffii*
DROWI	*Drosophila willistoni*	SHEEP	*Ovis aries*
EMIHU	*Emiliania huxleyi*	SPIPN	*Spizellomyces punctatus DAOM BR117*
ENTBH	*Enterocytozoon bieneusi (strain H348)*	STRMM	*Strigamia maritima*
FELCA	*Felis catus*	STRPU	*Strongylocentrotus purpuratus*
FICAL	*Ficedula albicollis*	STRRB	*Strongyloides ratti*
GASAC	*Gasterosteus aculeatus*	STYLE	*Stylonychia lemnae*
GIAIN	*Giardia intestinalis*	TAKRU	*Takifugu rubripes*
GONPR	*Gonapodya prolifera JEL478*	TETNG	*Tetraodon nigroviridis*
GORGO	*Gorilla gorilla gorilla*	TETTS	*Tetrahymena thermophila (strain SB210)*
GUITH	*Guillardia theta CCMP2712*	TETUR	*Tetranychus urticae*
HAMHA	*Hammondia hammondi*	THAOC	*Thalassiosira oceanica*
HELRO	*Helobdella robusta*	THAPS	*Thalassiosira pseudonana*
HETGA	*Heterocephalus glaber*	THETB	*Thecamonas trahens ATCC 50062*
HORSE	*Equus caballus*	TOXGV	*Toxoplasma gondii (strain ATCC 50861 / VEG)*
HUMAN	*Homo sapiens*	TRIAD	*Trichoplax adhaerens*
ICHMG	*Ichthyophthirius multifiliis (strain G5)*	TRICA	*Tribolium castaneum*
ICTTR	*Ictidomys tridecemlineatus*	TRIVA	*Trichomonas vaginalis*
IXOSC	*Ixodes scapularis*	TRYCC	*Trypanosoma cruzi (strain CL Brener)*
LATCH	*Latimeria chalumnae*	TRYCR	*Trypanosoma cruzi Dm28c*
LEIBR	*Leishmania braziliensis*	TRYRA	*Trypanosoma rangeli SC58*
LEIIN	*Leishmania infantum*	VITBC	*Vitrella brassicaformis (strain CCMP3155)*
LEIMA	*Leishmania major*	XENTR	*Xenopus tropicalis*
LEPOC	*Lepisosteus oculatus*	XIPMA	*Xiphophorus maculatus*
LEPSE	*Leptomonas seymouri*	ZOONE	*Zootermopsis nevadensis*
LOTGI	*Lottia gigantea*	9EIME	*Eimeria mitis*
LOXAF	*Loxodonta africana*	9EUKA	*Spironucleus salmonicida*
LUCCU	*Lucilia cuprina*	9FUNG	*Rozella allomycis CSF55*
MACFA	*Macaca fascicularis*	9MICR	*Anncaliia algerae PRA109*
MACMU	*Macaca mulatta*	9SPIT	*Oxytricha trifallax*
MONBE	*Monosiga brevicollis*	9STRA	*Saprolegnia diclina VS20/ Albugo candida/ Aphanomyces invadans/ Aphanomyces astaci*
MONDO	*Monodelphis domestica*	9TRYP	*Phytomonas sp. isolate Hart1/ Phytomonas sp. isolate EM1/ Leptomonas / Strigomonas culicis pyrrhocoris/ Angomonas deanei*
MOUSE	*Mus musculus*		

Table S1. Eukaryotic species with orthologous sequences in the G3PO benchmark.

BBS family	Human reference protein						No. of orthologous sequences
	Uniprot name	Uniprot Access	Length (AA)	No. of domains	No. of repeats	No. of low complexity regions	
BBS 1	BBS1_HUMAN	Q8NFJ9	593	1	0	7	127
BBS 2	BBS2_HUMAN	Q9BXC9	721	3	0	3	119
BBS 3	ARL6_HUMAN	Q9H0F7	186	1	0	1	119
BBS 4	BBS4_HUMAN	Q96RK4	519	10	10	5	130
BBS 5	BBS5_HUMAN	Q8N3I7	341	1	0	0	125
BBS 6	MKKS_HUMAN	Q9NPJ1	570	1	0	0	54
BBS 7	BBS7_HUMAN	Q8IWZ6	715	2	0	2	117
BBS 8	TTC8_HUMAN	Q8TAM2	541	8	8	5	138
BBS 9	PTHB1_HUMAN	Q3SYG4	887	2	0	5	131
BBS 10	BBS10_HUMAN	Q8TAM1	723	1	0	4	37
BBS 11	TRI32_HUMAN	Q13049	648	6	5	3	40
BBS 12	BBS12_HUMAN	Q6ZW61	710	1	0	3	44
BBS 13	MKS1_HUMAN	Q9NXB0	559	1	0	6	90
BBS 15	FRITZ_HUMAN	O95876	746	3	2	6	71
BBS 16	SDCG8_HUMAN	Q86SQ7	713	1	0	8	54
BBS 17	LZTL1_HUMAN	Q9NQ48	299	1	0	4	79
BBS 18	BBIP1_HUMAN	A8MTZ0	92	1	0	2	36
BBS 19	IFT27_HUMAN	Q9BW83	181	1	0	0	85
BBS 20	IFT74_HUMAN	Q96LB3	600	0	0	4	131
BBS 21	CH037_HUMAN	Q96NL8	207	1	0	4	66

Table S2. Bardet-Biedl Syndrome (BBS) protein families used in the benchmark. 'Low complexity regions' include predicted low complexity, disordered and coiled coil regions.

<table>
<tr><th colspan="4">Clade</th><th>No. of species</th><th>No. of sequences</th><th>No. of Confirmed sequences</th></tr>
<tr><td rowspan="10">Opisthokonta</td><td rowspan="8">Metazoa</td><td rowspan="2">Chordata</td><td>Craniata</td><td>46</td><td>766</td><td>503</td></tr>
<tr><td>Tunicata</td><td>2</td><td>25</td><td>9</td></tr>
<tr><td colspan="2">Mollusca</td><td>2</td><td>33</td><td>12</td></tr>
<tr><td colspan="2">Platyhelminthes</td><td>1</td><td>10</td><td>1</td></tr>
<tr><td colspan="2">Panarthropoda</td><td>29</td><td>264</td><td>96</td></tr>
<tr><td colspan="2">Nematoda</td><td>8</td><td>71</td><td>28</td></tr>
<tr><td colspan="2">Cnidaria</td><td>1</td><td>18</td><td>8</td></tr>
<tr><td colspan="2">Others Metazoa</td><td>3</td><td>49</td><td>18</td></tr>
<tr><td colspan="3">Fungi</td><td>8</td><td>25</td><td>11</td></tr>
<tr><td colspan="3">Choanoflagellida</td><td>2</td><td>22</td><td>5</td></tr>
<tr><td colspan="4">Stramenopila</td><td>12</td><td>172</td><td>88</td></tr>
<tr><td colspan="4">Euglenozoa</td><td>9</td><td>149</td><td>60</td></tr>
<tr><td colspan="4">Viridiplantae</td><td>4</td><td>12</td><td>4</td></tr>
<tr><td colspan="4">Alveolata</td><td>11</td><td>99</td><td>24</td></tr>
<tr><td colspan="4">Rhizaria</td><td>2</td><td>21</td><td>5</td></tr>
<tr><td colspan="4">Others</td><td>7</td><td>57</td><td>17</td></tr>
</table>

Table S3. Phylogenetic distribution of benchmark sequences. 'Others Metazoa' contains 3 species that were not classified in one of the main groups, and 'Others' contains 7 unicellular eukaryote species.

Uniprot Species identifier	No. of sequences	No. of Unconfirmed sequences	% Unconfirmed sequences	Uniprot Species identifier	No. of sequences	No. of Unconfirmed sequences	% Unconfirmed sequences
HUMAN	20	0	0	FICAL	17	10	59
HORSE	20	3	15	DROSE	10	6	60
RAT	20	3	15	LEIBR	10	6	60
CALJA	19	3	16	THECL	10	6	60
MOUSE	19	3	16	TRYB2	10	6	60
RABIT	18	3	17	TRYRA	10	6	60
BRUPA	10	2	20	CHLRE	13	8	62
CHLSB	20	4	20	PLABS	13	8	62
PONAB	20	4	20	PYTUL	13	8	62
LOXAF	19	4	21	9HYME	45	28	62
NOMLE	19	4	21	PELSI	19	12	63
XIPMA	19	4	21	ACREC	11	7	64
MACFA	18	4	22	CAERE	11	7	64
BOVIN	21	5	24	CULQU	11	7	64
GORGO	19	5	26	ENTVE	11	7	64
MACMU	19	5	26	PHYNI	11	7	64
TETNG	15	4	27	SALR5	11	7	64
ACYPI	10	3	30	VITBC	14	9	64
PHYIT	13	4	31	9BILA	38	25	66
PHYSP	13	4	31	LEIIN	12	8	67
AILME	19	6	32	MICPC	12	8	67
OTOGA	19	6	32	STYLE	12	8	67
PIG	19	6	32	9TRYP	54	36	67
POEFO	19	6	32	ASTMX	19	13	68
MUSPF	18	6	33	ALLMI	16	11	69
PHYPR	24	8	33	AEDAE	10	7	70
CAVPO	20	7	35	PHYRM	10	7	70
ICTTR	17	6	35	CRAGI	17	12	71
CANLF	19	7	37	STRMM	14	10	71
TAKRU	16	6	38	CAEBE	11	8	73
TRIAD	16	6	38	LEIMA	11	8	73
TRYCR	16	6	38	NASVI	11	8	73
FELCA	18	7	39	PEDHC	11	8	73
FUKDA	18	7	39	MYOBR	19	14	74
PANTR	18	7	39	CHEMY	12	9	75
SARHA	18	7	39	CIOSA	12	9	75
9STRA	46	18	39	MICCC	12	9	75
STREA	10	4	40	NAEGR	12	9	75
BRUMA	12	5	42	TOXCA	12	9	75
PHYPN	12	5	42	STRPU	20	15	75
PAPAN	19	8	42	9SPIT	13	10	77
DANRE	21	9	43	HAECO	13	10	77

GASAC	16	7	44	HELRO	13	10	77
ORYLA	16	7	44	NIPBR	13	10	77
ANOCA	18	8	44	AMPQE	18	14	78
SHEEP	18	8	44	AMAAE	14	11	79
ORENI	20	9	45	PARTE	14	11	79
LOALO	11	5	45	TUPCH	14	11	79
TRICA	11	5	45	ANOGA	10	8	80
TRIVA	13	6	46	ASCSU	10	8	80
HETGA	17	8	47	CAEBR	10	8	80
MONDO	19	9	47	PAPMA	10	8	80
ORNAN	19	9	47	PSEPJ	10	8	80
PARTI	10	5	50	TETUR	10	8	80
CHICK	16	8	50	OIKDI	11	9	82
TRYCC	18	9	50	RHOPR	11	9	82
XENTR	20	10	50	ECTSI	13	11	85
MYODS	15	8	53	9TREM	18	16	89
9EUKA	13	7	54	9TELE	19	17	89
CIOIN	13	7	54	9CHLO	10	9	90
ZOONE	13	7	54	CLOSI	10	9	90
ATTCE	11	6	55	DRAME	10	9	90
CAMFO	11	6	55	NECAM	10	9	90
LEPSE	11	6	55	SCHHA	10	9	90
ONCVO	11	6	55	SCHMA	10	9	90
VOLCA	11	6	55	CAMFR	11	10	91
BRAFL	20	11	55	DANPL	11	10	91
LEPOC	20	11	55	DAPPU	11	10	91
MYOLU	18	10	56	IXOSC	11	10	91
NEMVE	18	10	56	MONBE	11	10	91
LOTGI	16	9	56	PAPXU	12	11	92
SAPPC	16	9	56	DENPD	10	10	100
LATCH	19	11	58	PERM5	10	10	100
PTEAL	19	11	58	SARSC	10	10	100
GUITH	12	7	58	AURAN	11	11	100
STRER	12	7	58	OPHHA	16	16	100
TETTS	12	7	58				

Table S4. Species with at least 10 sequences in the benchmark, ranked by the percentage of Unconfirmed sequences identified.

			**DNA sequence**		**Exon map**			**Protein sequence**
		No. of sequences	Mean gene length	Mean %GC	Mean no. of exons	Mean exon length	Mean intron length	Mean protein length
	All	283	95584	37.4	14	186	6269	551
	Confirmed	133	95533	39.81	14	179	6047	515

With UDT regions	Unconfirmed	150	95629	39.76	14	192	6466	582
Without UDT regions	All	1510	15934	44.2	7.6	563	1161	514
	Confirmed	756	18367	43.9	8.0	556	1438	482
	Unconfirmed	754	13496	44.6	7.2	570	883	546

Table S5. Comparison of the 283 gene sequences with undetermined (UDT) regions and 1510 sequences without UDT regions for both Confirmed and Unconfirmed sequences.

	**Nucleotide level**			**Exon level**						**Protein level**	
	Sn	Sp	F1	Sn	Sp	ME	WE	5' (first exon)	3' (last exon)	% Identity	Perfect (100%)
Augustus	0.51	0.58	0.52	0.27	0.30	0.65	0.62	31.0 (27.6)	31.2 (32.5)	75.39	209
Genscan	0.50	0.57	0.51	0.23	0.28	0.74	0.69	31.5 (25.6)	33.3 (24.0)	71.74	135
GeneID	0.38	0.52	0.40	0.14	0.19	0.85	0.79	24.6 (19.7)	27.0 (18.0)	52.57	91
GlimmerHMM	0.74	0.43	0.45	0.18	0.22	0.81	0.75	24.8 (22.7)	31.4 (23.2)	60.06	136
Snap	0.38	0.45	0.39	0.15	0.18	0.67	0.64	20.5 (19.1)	21.8 (20.5)	46.60	112

Table S6. Overall performance the 5 gene prediction programs, using the 889 Confirmed sequences. Sn=sensitivity; Sp=specificity; F1=F1 score; ME=Missing Exons; WE=Wrong Exons; 5'=Percentage of correctly predicted 5' exon boundaries (first exon=Percentage of correctly predicted 5' boundaries of first exons only); 3'=Percentage of correctly predicted 3' exon boundaries (last exon=Percentage of correctly predicted 3' boundaries of the last exons only). % Identity indicates the average sequence identity observed between the predicted proteins and the benchmark sequences. 'Perfect' indicates proteins predicted with 100% identity compared to the benchmark sequence.

**Program**	**CPU time for gene sequence +150bp upstream and downstream (seconds)**	**CPU time for gene sequence +10Kb upstream and downstream (seconds)**
Augustus	1826	4172
Genscan	484	897
GeneID	196	260
GlimmerHMM	540	698
Snap	266	443

Table S7. Total time required to process the 1793 genomic sequences covering the gene region with 150bp (total length = 51,699,512 nucleotides) and with 10Kb upstream/downstream flanking sequences (total length = 86,970,612 nucleotides).

Augustus

	**Nucleotide level**			**Exon level**				**Protein level**	
	Sn	Sp	F1	Sn	Sp	ME	WE	% Identity	Perfect (100%)
10Kb	0.50	0.61	0.53	0.25	0.28	0.67	0.62	67.71	172
8Kb	0.51	0.60	0.53	0.26	0.28	0.67	0.62	68.25	172
6Kb	0.51	0.60	0.53	0.26	0.28	0.67	0.62	68.68	176
4Kb	0.51	0.60	0.53	0.26	0.28	0.67	0.63	69.50	177
2Kb	0.51	0.59	0.53	0.26	0.28	0.67	0.62	72.43	186
150b	0.51	0.58	0.52	0.27	0.30	0.65	0.62	75.39	209

Genscan

	Nucleotide level			Exon level				Protein level	
	Sn	Sp	F1	Sn	Sp	ME	WE	% Identity	Perfect (100%)
10Kb	0.50	0.61	0.53	0.20	0.24	0.77	0.72	58.79	106
8Kb	0.50	0.61	0.52	0.20	0.24	0.77	0.72	59.35	106
6Kb	0.50	0.60	0.52	0.20	0.24	0.77	0.72	59.97	105
4Kb	0.50	0.60	0.52	0.20	0.24	0.77	0.72	61.11	104
2Kb	0.50	0.59	0.52	0.20	0.24	0.77	0.72	64.20	103
150b	0.50	0.57	0.51	0.23	0.28	0.74	0.69	71.74	135

GeneID

	Nucleotide level			Exon level				Protein level	
	Sn	Sp	F1	Sn	Sp	ME	WE	% Identity	Perfect (100%)
10Kb	0.38	0.56	0.42	0.13	0.17	0.87	0.76	48.61	74
8Kb	0.38	0.56	0.42	0.13	0.17	0.87	0.76	48.74	75
6Kb	0.38	0.56	0.42	0.13	0.17	0.87	0.76	49.06	75
4Kb	0.38	0.56	0.42	0.13	0.17	0.87	0.76	49.61	75
2Kb	0.38	0.55	0.41	0.13	0.17	0.87	0.77	50.31	76
150b	0.38	0.52	0.40	0.14	0.19	0.85	0.79	52.57	91

GlimmerHMM

	Nucleotide level			Exon level				Protein level	
	Sn	Sp	F1	Sn	Sp	ME	WE	% Identity	Perfect (100%)
10Kb	0.83	0.45	0.49	0.19	0.23	0.81	0.74	59.25	139
8Kb	0.83	0.45	0.49	0.19	0.23	0.81	0.74	59.59	139
6Kb	0.82	0.45	0.49	0.19	0.23	0.80	0.74	59.81	141
4Kb	0.81	0.45	0.49	0.19	0.23	0.80	0.74	60.32	141
2Kb	0.78	0.44	0.48	0.18	0.22	0.80	0.75	60.21	138
150b	0.74	0.43	0.45	0.18	0.22	0.81	0.75	60.06	136

Snap

	Nucleotide level			Exon level				Protein level	
	Sn	Sp	F1	Sn	Sp	ME	WE	% Identity	Perfect (100%)
10Kb	0.38	0.53	0.40	0.14	0.17	0.72	0.67	45.52	109
8Kb	0.38	0.53	0.40	0.15	0.18	0.71	0.67	45.68	111
6Kb	0.38	0.52	0.40	0.15	0.18	0.71	0.67	45.58	112
4Kb	0.38	0.52	0.40	0.15	0.18	0.70	0.66	46.14	113
2Kb	0.38	0.49	0.40	0.15	0.18	0.69	0.66	46.21	113
150b	0.38	0.45	0.39	0.15	0.18	0.67	0.64	46.60	112

Table S8. Effect of the genomic context based on the different lengths of upstream/downstream flanking genomic sequences on the performance of the 5 gene prediction programs at the nucleotide, exon and protein levels, using 889 Confirmed sequences. Sn=sensitivity; Sp=specificity; F1=F1 score; ME=Missing Exons; WE=Wrong Exons. %Identity indicates the average sequence identity observed between the predicted proteins and the benchmark sequences. 'Perfect' indicates proteins predicted with 100% identity compared to the benchmark sequence.

542 Metazoan sequences without UDT regions

	**Nucleotide level**			**Exon level**						**Protein level**	
	Sn	Sp	F1	Sn	Sp	ME	WE	5’ (start)	3’ (stop)	% Identity	Perfect (100%)
Augustus	0.44	0.55	0.47	0.23	0.26	0.69	0.64	32.1 (21.6)	33.2 (26.2)	74.44	94
Genscan	0.45	0.57	0.48	0.19	0.24	0.79	0.73	33.1 (14.9)	33.4 (24.7)	67.13	46
GeneID	0.34	0.52	0.38	0.14	0.18	0.86	0.77	25.7 (14.8)	26.5 (15.3)	52.26	41
Glimmer HMM	0.77	0.38	0.42	0.14	0.18	0.85	0.79	25.8 (17.7)	26.3 (19.0)	59.36	57
Snap	0.33	0.46	0.35	0.14	0.17	0.73	0.68	23.5 (17.5)	25.4 (15.5)	44.20	51

133 Metazoan sequences with UDT regions

	**Nucleotide level**			**Exon level**						**Protein level**	
	Sn	Sp	F1	Sn	Sp	ME	WE	5’ (start)	3’ (stop)	% Identity	Perfect (100%)
Augustus	0.27	0.35	0.27	0.22	0.26	0.71	0.64	33.9 (20.3)	34.0 (18.0)	66.87	4
Genscan	0.29	0.27	0.25	0.17	0.22	0.82	0.76	28.3 (12.0)	29.7 (7.5)	52.88	3
GeneID	0.19	0.22	0.17	0.11	0.16	0.89	0.82	22.9 (9.0)	24.8 (3.8)	34.91	0
Glimmer HMM	0.71	0.08	0.12	0.12	0.17	0.88	0.82	20.8 (6.0)	22.3 (5.3)	42.97	5
Snap	0.15	0.19	0.14	0.07	0.09	0.72	0.70	12.7 (12.8)	14.9 (0.0)	25.19	0

Table S9. Performance of the 5 gene prediction programs, using all 542 metazoan sequences without undetermined (UDT) regions in reference genes and all 133 metazoans with UDT regions in reference gene. Sn=sensitivity; Sp=specificity; F1=F1 score; ME=Missing Exons; WE=Wrong Exons; 5’=Percentage of correctly predicted 5’ exon boundaries (first exon=Percentage of correctly predicted 5’ boundaries of first exons only); 3’=Percentage of correctly predicted 3’ exon boundaries (last exon=Percentage of correctly predicted 3’ boundaries of the last exons only). % Identity indicates the average sequence identity observed between the predicted proteins and the benchmark sequences. ‘Perfect’ indicates proteins predicted with 100% identity compared to the benchmark sequence.

A)

	No. of exons in G3PO	Augustus	Genscan	GeneID	GlimmerHMM	Snap
[0-50]	374	18.4%	13.6%	12.3%	6.7%	9.4%
]50-100]	1919	24.4%	20.0%	11.8%	12.6%	11.6%
]100-150]	1846	25.6%	22.4%	12.7%	13.3%	12.7%
]150-200]	792	25.1%	21.3%	15.4%	15.0%	13.9%
>200	1180	17.3%	15.2%	10.9%	14.0%	12.5%
Total exons	6111	23.1%	19.6%	12.4%	13.0%	12.3%

B)

		Correct	Wrong	Wrong			
				Both	5’	3’	Fusion
Augustus	[0-50]	69	187	147	24	16	0
	]50-100]	468	795	700	45	50	0
	]100-150]	473	871	739	65	67	0
	]150-200]	199	371	306	38	26	1
	>200	204	343	145	104	80	14
Genscan	[0-50]	51	116	98	8	10	0
	]50-100]	384	704	619	44	41	0
	]100-150]	413	916	776	74	66	0
	]150-200]	169	455	365	48	39	3
	>200	180	448	226	134	73	15
GeneID	[0-50]	46	359	296	34	29	0
	]50-100]	226	595	518	38	39	0
	]100-150]	234	625	520	54	51	0
	]150-200]	122	304	242	26	36	0
	>200	129	387	207	102	74	4
Glimmer HMM	[0-50]	25	177	153	11	13	0
	]50-100]	241	749	661	41	47	0
	]100-150]	245	709	606	57	46	0
	]150-200]	119	327	252	37	37	1
	>200	165	392	209	86	90	7
SNAP	[0-50]	35	292	261	13	18	0
	]50-100]	222	594	501	46	47	0
	]100-150]	235	514	408	49	57	0
	]150-200]	110	259	187	38	33	1
	>200	148	304	154	78	65	7

Table S10. Effect of exon length on exon prediction quality. A) Proportion of all benchmark exons correctly predicted depending on the exon length. B) Number of internal exons predicted correctly, with either the 5’ or 3’ exon boundaries correct, or with both sites wrongly predicted, for each of the five programs.

	Protein length (amino acids)	% Identity	Perfect (100%)
Augustus	<100	57.45	4
	100-300	67.37	40
	300-550	75.27	58
	550-650	81.78	55
	>650	70.04	25
Genscan	<100	60.65	1
	100-300	52.56	13
	300-550	62.77	27
	550-650	78.78	42
	>650	70.29	17
GeneID	<100	51.31	2
	100-300	44.06	6
	300-550	54.47	21
	550-650	61.74	34
	>650	51.10	13
Glimmer HMM	<100	68.30	5
	100-300	58.03	27
	300-550	63.21	38
	550-650	70.09	48
	>650	60.32	15
Snap	<100	34.29	3
	100-300	47.58	16
	300-550	57.66	42
	550-650	56.10	39
	>650	38.85	13

Table S11. Effect of protein length on prediction accuracy at the protein level. %Identity indicates the average sequence identity observed between the predicted proteins and the benchmark sequences (Confirmed without UDT regions). 'Perfect' indicates proteins predicted with 100% identity compared to the benchmark sequence.

Clade				Augustus		Genscan		GeneID		GlimmerHMM		Snap	
				% Identity	Perfect (100%)	% Identity	Perfect (100%)	% Identity	Perfect (100%)	% Identity	Perfect (100%)	% Identity	Perfect (100%)
Opisthokonta	Metazoa	Chordata	Craniata	72.23	44	69.71	31	49.22	19	55.86	23	33.03	16
			Tunicata	85.90	1	54.44	0	59.22	0	27.38	0	77.87	0
		Mollusca		89.64	2	68.22	1	30.60	1	70.80	2	56.14	1
		Panarthropoda		82.60	30	69.11	13	66.57	14	69.18	15	73.82	21
		Nematoda		78.38	15	47.16	1	76.35	7	89.19	15	81.80	12
		Cnidaria		32.34	1	50.98	0	30.11	0	61.43	2	39.35	0
	Fungi			20.68	0	28.93	1	23.54	0	57.82	4	50.27	0
	Choanoflagellida			20.57	0	66.07	0	38.06	0	26.44	0	15.62	0
Stramenopila				67.73	21	70.33	22	59.66	16	76.55	19	76.08	28
Euglenozoa				97.65	53	77.02	29	77.58	18	97.36	48	84.55	33

Viridiplantae	94.77	2	43.57	0	73.50	0	62.34	0	48.46	0
Alveolata	47.89	5	26.79	0	17.52	0	26.47	1	16.55	0
Rhizaria	56.16	0	44.74	0	47.03	0	62.28	0	54.91	0
Others	71.13	8	54.79	2	23.01	2	52.85	4	38.81	2

Table S12. Performance of the 5 gene prediction programs for sequences from different clades. The 'Others' group contains the Apusozoa, Cryptophyta, Diplomonadida, Haptophyceae, Heterolobosea, Parabasalia clades, as well as Placozoa, Annelida and urchin. %Identity indicates the average sequence identity observed between the predicted proteins and the benchmark sequences (Confirmed without UDT regions). 'Perfect' indicates proteins predicted with 100% identity compared to the benchmark sequence.

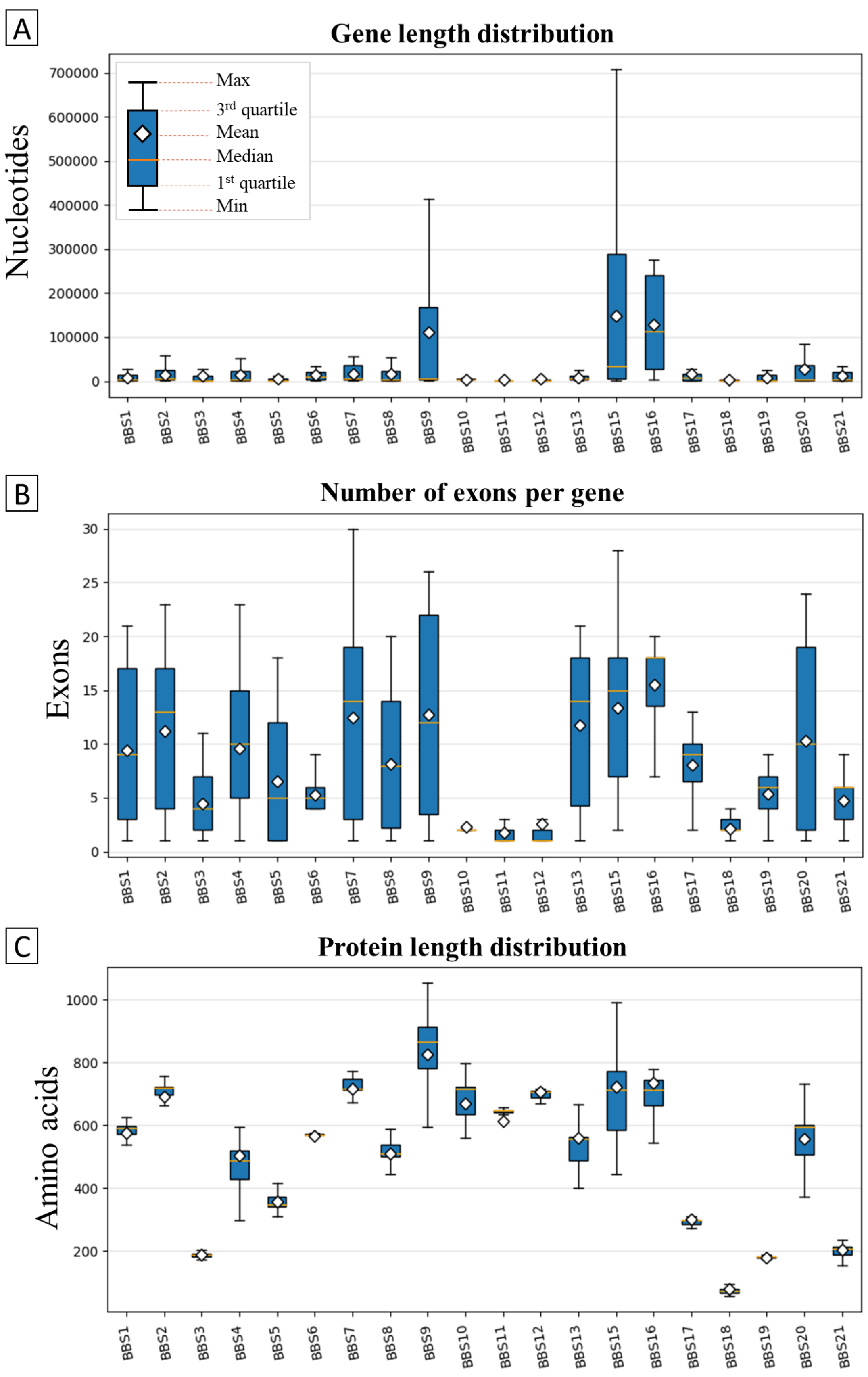

Figure S1. Distribution of A) gene length, B) number of exons and C) protein length for each orthologous protein family.

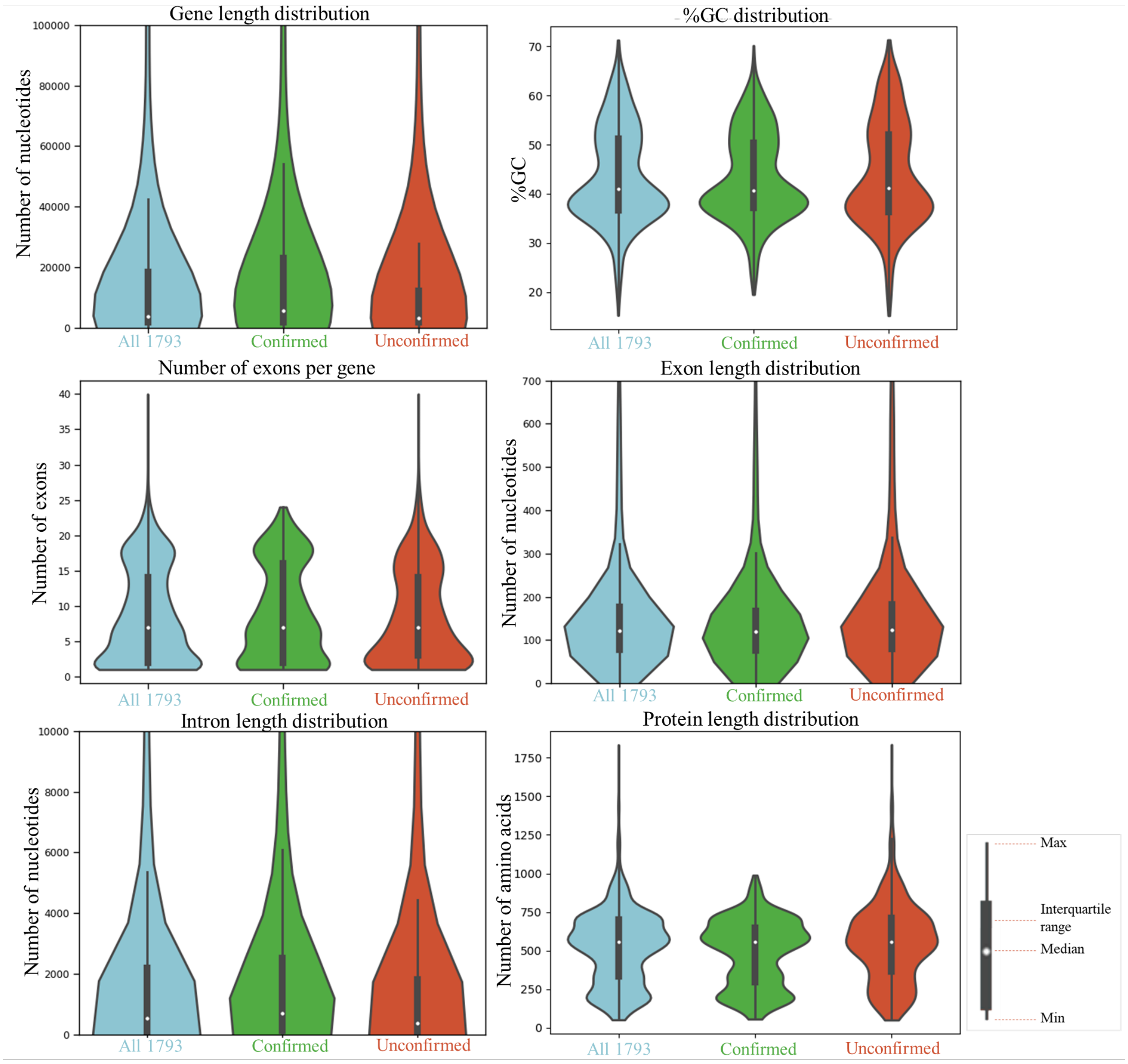

Figure S2. Main characteristics of the 1793 test cases in the benchmark for All sequences, Confirmed and Unconfirmed sequences only.

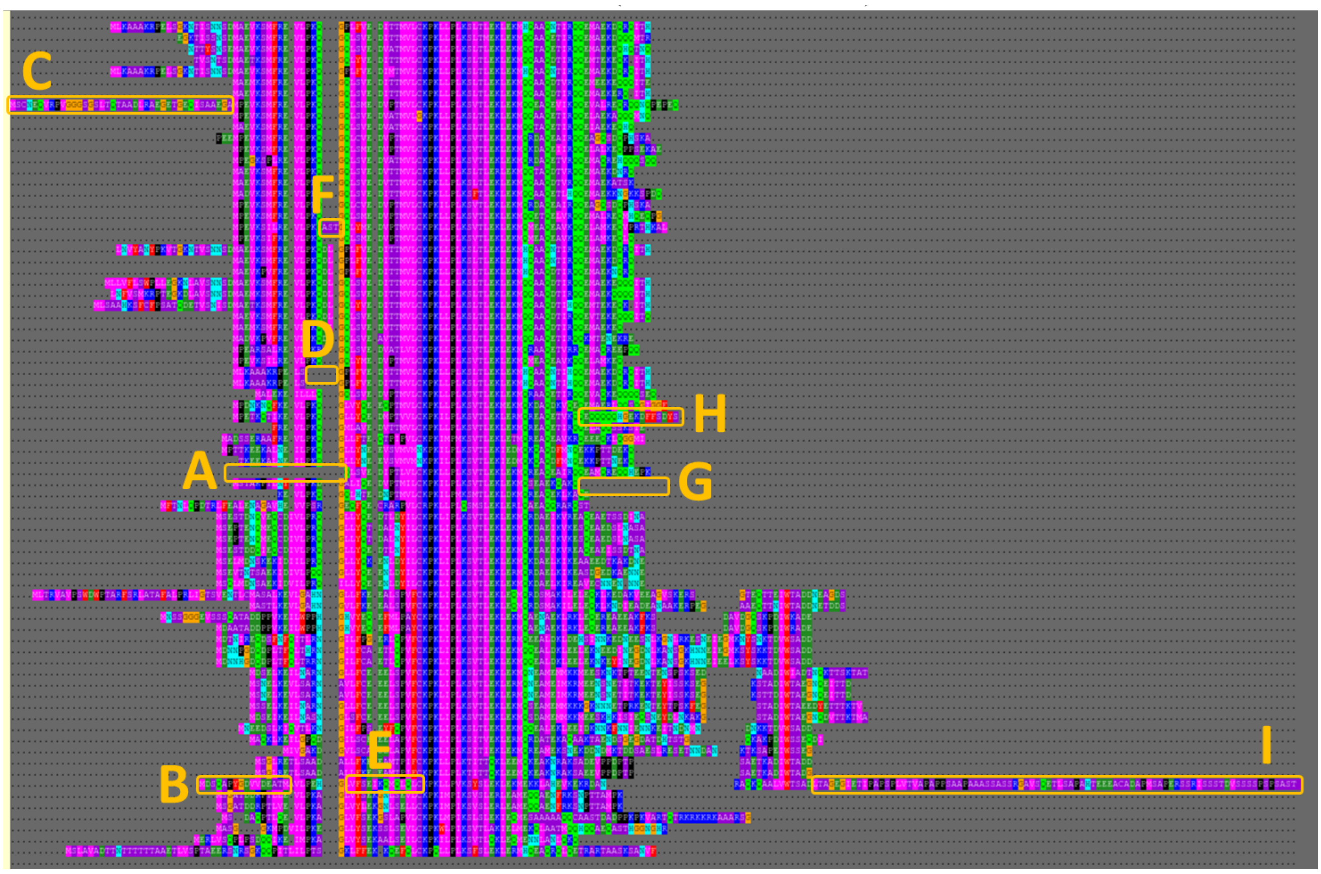

Figure S3. Schematic view of an MSA, showing the 9 categories of sequence errors, highlighted by orange boxes. **A**: N-terminal deletion **B**: N-terminal mismatched segment **C**: N-terminal insertion **D**: Internal deletion **E**: Internal mismatched segment **F**: Internal insertion **G**: C-terminal deletion **H**: C-terminal mismatched segment **I**: C-terminal insertion.

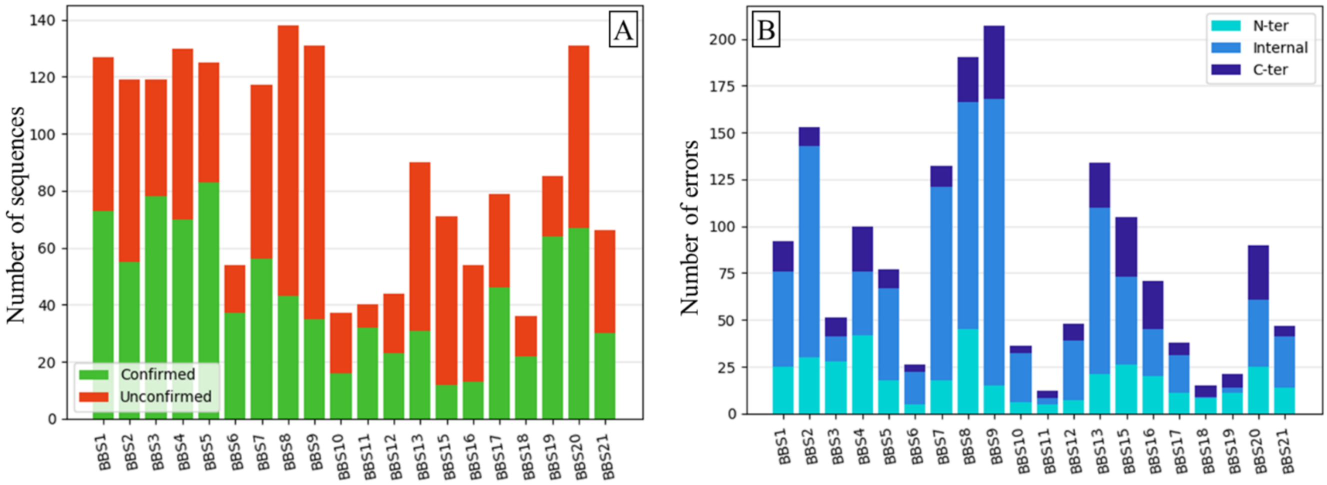

Figure S4. A) Number of Confirmed and Unconfirmed sequences in each orthologous protein family. B) Number and types of error in each orthologous protein family.

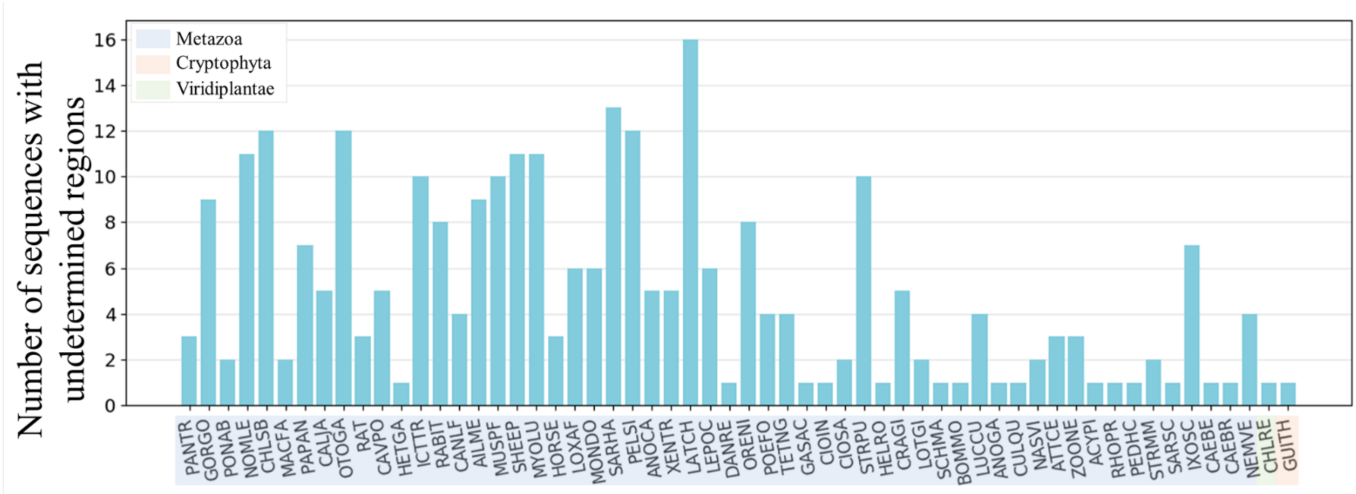

Figure S5. Number of sequences with undetermined (UDT) regions in each species.

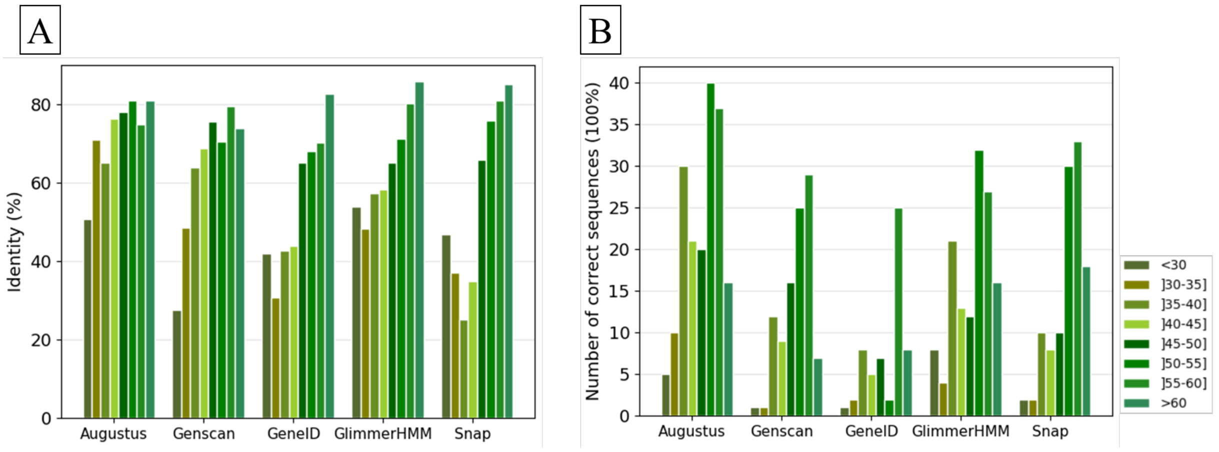

Figure S6. Effect of the %GC content of the gene on prediction accuracy: A) average percent identity between the predicted and the benchmark protein sequences, B) number of proteins perfectly predicted with 100% sequence identity.

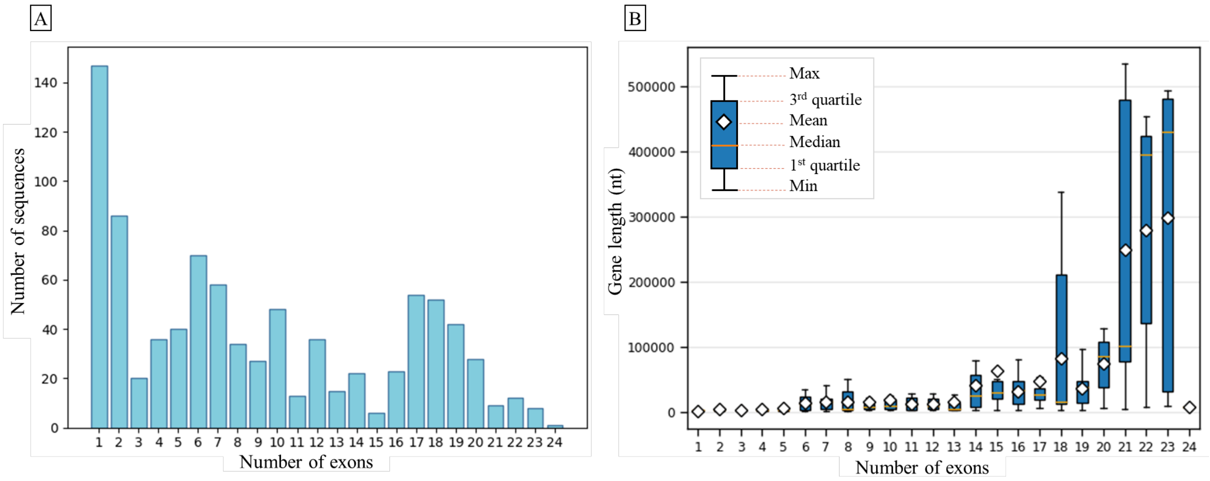

Figure S7. A) Number of Confirmed sequences with exon count ranging from 1 to 24. B) Distribution of gene lengths for sequences with exon count ranging from 1 to 24. 45% of

sequences have 1 to 6 exons. A second peak is observed at 17-19 exons, which correspond mostly to sequences from higher metazoans. The average gene sequence length increases with the complexity of the exon map (correlation=0.749, p-value=1.833e^{-160}).

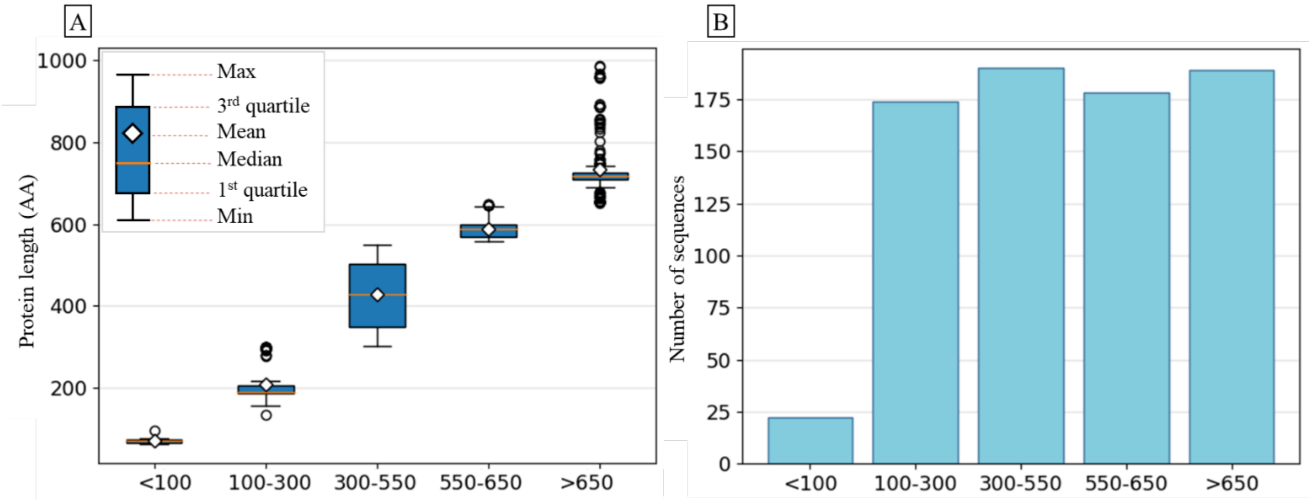

Figure S8. A) Distribution of protein lengths for Confirmed sequences. B) Number of Confirmed sequences with different protein lengths.

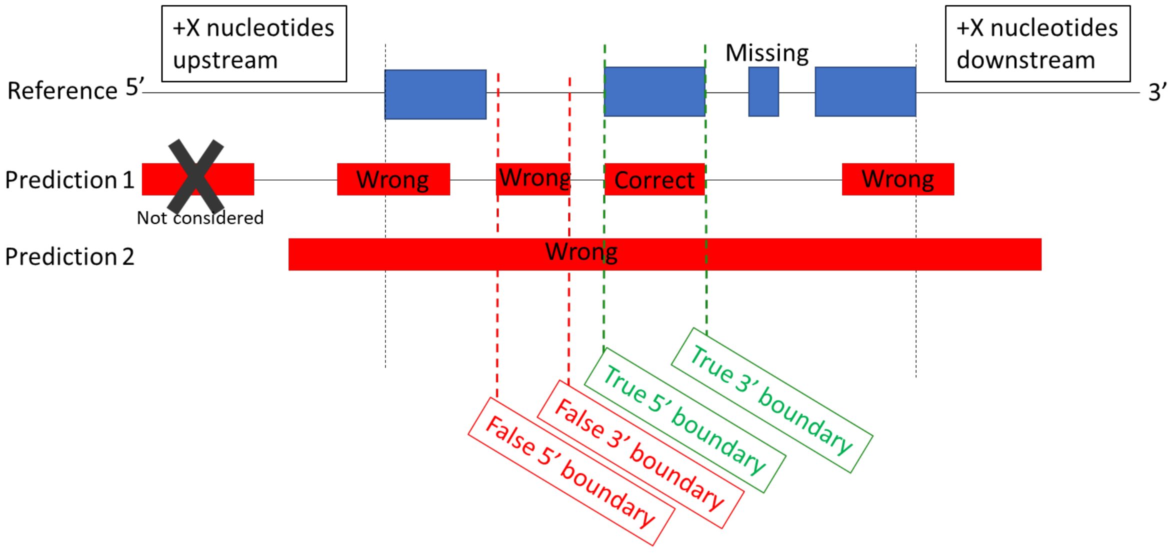

Figure S9. Evaluation metrics at the exon level.
